# Prevalence of trace gas-oxidizing soil bacteria increases with radial distance from Polloquere hot spring within a high-elevation Andean cold desert

**DOI:** 10.1093/ismejo/wrae062

**Published:** 2024-04-16

**Authors:** Zachary K Garvin, Sebastián R Abades, Nicole Trefault, Fernando D Alfaro, Katie Sipes, Karen G Lloyd, Tullis C Onstott

**Affiliations:** Department of Geosciences, Princeton University, Princeton, NJ 08544, United States; GEMA Center for Genomics, Ecology and Environment, Faculty of Interdisciplinary Studies, Universidad Mayor, 8580745, Santiago, Chile; GEMA Center for Genomics, Ecology and Environment, Faculty of Interdisciplinary Studies, Universidad Mayor, 8580745, Santiago, Chile; GEMA Center for Genomics, Ecology and Environment, Faculty of Interdisciplinary Studies, Universidad Mayor, 8580745, Santiago, Chile; Department of Microbiology, University of Tennessee, Knoxville, TN 37996, United States; Department of Environmental Science, Aarhus University, 4000, Roskilde, Denmark; Department of Microbiology, University of Tennessee, Knoxville, TN 37996, United States; Department of Geosciences, Princeton University, Princeton, NJ 08544, United States

**Keywords:** geomicrobiology, microbial ecology, trace gas, metagenomics

## Abstract

High-elevation arid regions harbor microbial communities reliant on metabolic niches and flexibility to survive under biologically stressful conditions, including nutrient limitation that necessitates the utilization of atmospheric trace gases as electron donors. Geothermal springs present “oases” of microbial activity, diversity, and abundance by delivering water and substrates, including reduced gases. However, it is unknown whether these springs exhibit a gradient of effects, increasing their impact on trace gas-oxidizers in the surrounding soils. We assessed whether proximity to Polloquere, a high-altitude geothermal spring in an Andean salt flat, alters the diversity and metabolic structure of nearby soil bacterial populations compared to the surrounding cold desert. Recovered DNA and metagenomic analyses indicate that the spring represents an oasis for microbes in this challenging environment, supporting greater biomass with more diverse metabolic functions in proximal soils that declines sharply with radial distance from the spring. Despite the sharp decrease in biomass, potential rates of atmospheric hydrogen (H_2_) and carbon monoxide (CO) uptake increase away from the spring. Kinetic estimates suggest this activity is due to high-affinity trace gas consumption, likely as a survival strategy for energy/carbon acquisition. These results demonstrate that Polloquere regulates a gradient of diverse microbial communities and metabolisms, culminating in increased activity of trace gas-oxidizers as the influence of the spring yields to that of the regional salt flat environment. This suggests the spring holds local importance within the context of the broader salt flat and potentially represents a model ecosystem for other geothermal systems in high-altitude desert environments.

## Introduction

Arid soil environments constrain biological activity mostly due to low water availability [1–3], along with drastic temperature fluctuations and high ultraviolet radiation flux [4]. Thus, microbial communities in these regions are often sparse with reduced cell density. Microbial colonization of arid soils is typically patchy, forming assemblages like endolithic communities or biocrusts in environments where substrate availability is higher and the harsh conditions are partially relieved or locally mitigated [4–6].

Although many arid regions contain biocrusts and mats supported by photoautotrophy, the oxidation of trace gas species has been identified as another common metabolic strategy in dry, oligotrophic soils [7–14]. High-affinity oxidation of atmospheric hydrogen (H_2_), carbon monoxide (CO), and methane (CH_4_) is prevalent globally in a variety of soil environments, providing a supplemental source of electrons for aerobic respiration and carbon fixation for bacteria [7]. The biological oxidation of trace gases is vital to biogeochemical cycles [15] and represents a significant sink of atmospheric gas that is increasingly considered in global models [16–18]. Experimental and genomic evidence of trace gas-oxidizing bacteria has been shown in diverse arid environments, including polar Arctic/Antarctic soils [10, 11, 13, 14, 19, 20] and hot deserts [8, 9, 12, 21, 22]. It is hypothesized that trace gas metabolisms are a survival mechanism for maintaining cellular function in these oligotrophic environments with scarce substrates and under conditions where alternative metabolisms may not be functional [23, 24]. Cultured representatives of trace gas-oxidizers show that these metabolisms occur during carbon limitation and biological stress [23, 25–28].

Trace gas oxidation has also been noted in volcanic and fumarolic environments [23, 29–31]. These features provide an elevated source of reduced gas species that are emitted into the atmosphere and carried through shallow subsurface channels. Hot springs could serve a similar role, as their geothermal fluid can contain high concentrations of reduced gas species that are transported to the surface from subsurface reservoirs when fed primarily from the vapor-phase of the boiling subsurface fluid [32–35]. Hot springs influenced by magmatic degassing are present globally in a variety of volcanic systems, including those in Iceland [36], New Zealand [37], Costa Rica [38], and Peru [39]. However, the influence of hot springs on their surrounding soil environments has not been fully explored. Hot springs and submarine volcanic systems host extremophilic organisms, including thermophilic archaea, that use the substrates dissolved in the geothermal fluids for growth [33, 40], whereas fumaroles can promote the establishment of photoautotrophic mats [41]. In arid regions, soils proximal to hot springs could represent a haven for microbial communities that take advantage of the abundant water and resources provided by the spring. Much less is known about whether reduced gases from the springs could stimulate atmospheric trace gas oxidation, resulting in higher rates of gas consumption and preferentially selecting for organisms adapted to the higher concentrations of these gases.

We selected the Polloquere hot spring in Salar de Surire of northern Chile to search for trace gas-oxidizing bacteria in the surrounding soils and determine the effects of hot spring proximity on soil microbial communities. The site, located within the Andean Central Volcanic Zone (ACVZ), has broad applicability to metabolic and community structures of soils in hot spring environments globally because it is heavily influenced by magmatic degassing. However, the ACVZ contains some attributes that make it unique relative to other widespread magmatically influenced zones. Specifically, Salar de Surire is one of several high-altitude, arid salt flats in the Andean Altiplano, located at 4200 m above sea level. The fault-bound basin is enclosed by sulfurous volcanoes and is presumed to be within a caldera [42]. Unlike many other volcanic zones distributed globally in a variety of tectonic settings [43], the Surire hydrothermal system is fueled by the underlying geothermal gradient from an ocean-continent convergent subduction boundary, and the surrounding soils are exposed to a suite of harsh conditions in contrast to the nutrient-rich environments often induced by local volcanism in wetter regions. The region is an endmember of an aridity, temperature, UV radiation, and elevation gradient extending from the Atacama Desert to the ACVZ. This salt flat experiences a multitude of extreme conditions, including aridity (35–391 mm yr^−1^; mean annual precipitation = 215 mm yr^−1^) with high evaporation rates (>75% of annual precipitation), low mean annual temperature (2.9°C) with large diurnal fluctuations (~15°C), and high UV flux (72 W m^−2^ UVA and 12 W m^−2^ UVB) [42, 44, 45]. Thus, the environment shares some similarities with lower-altitude deserts that have been previously found to host trace gas-oxidizing bacteria [9, 12, 21]. Though many of the hydrothermal features in Surire have dried out, a cluster of hot springs including Polloquere still persists within the southeastern edge of the salt flat. The springs are characterized by high concentrations of reduced gas species (including H_2_, H_2_S, and CH_4_) and consistent gas emission [42, 46], making Polloquere a prime candidate for studying the effects of these local hot springs on biological trace gas oxidation potential in the surrounding salt flat soils.

In this study, we quantified and compared atmospheric H_2_ and CO uptake rates across three soil transects surrounding Polloquere hot spring. We demonstrated an apparent increase in biological trace gas consumption with radial distance from the spring. Using metagenomic analyses, we corroborated these findings and showed that microbial communities differ in taxonomic diversity with an average dissimilarity of 93% depending on proximity to the spring with a clear shift toward microbes encoding for trace gas metabolisms at greater radial distances. Our results suggest that the influence of hot springs on local soil environments varies strongly with meters-scale spatial separation, thus establishing zones of microbial life adapted to the highly divergent conditions and progressively conforming to the conditions of the broader regional environment.

## Materials and methods

### Sample collection

**Figure 1:**
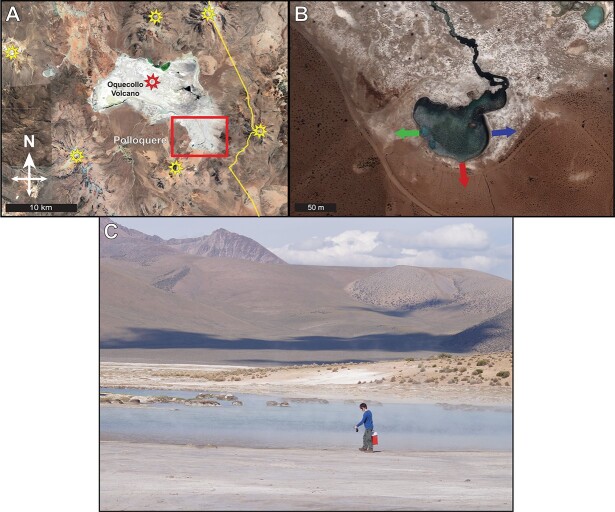
Polloquere hot spring field site and sampling locations; (A) map of Salar de Surire and the Polloquere geothermal region (red box) in northern Chile; yellow stars mark Plio-Pleistocene volcanoes with elevations ranging from 5000 to 6000 m above sea level; red star denotes the location of Oquecollo volcano at the center of the Salar basin; (B) enlarged satellite view of the Polloquere hot spring in the southeast of the Salar; arrows indicate the sampled soil transects (to scale; green: PQ1, red: PQ2, blue: PQ3); images courtesy of Google Earth and Maxar WV-03 © 2021; (C) field image of Polloquere hot spring and the surrounding sampled soils; the senior author is pictured for scale.

Soil, water, and atmospheric gas were sampled from Polloquere hot spring (18.9132°S, 68.9985°W) in the southeastern corner of Salar de Surire (Fig. 1A). Three water samples (50 ml each) were obtained from the spring’s western, eastern, and southern edges. Gas samples were collected from the steam emanating from the edge of the hot spring into preevacuated serum vials to estimate local enrichment of gases in the atmosphere above the adjacent soils. Soil was sampled from a series of three transects beginning at the edge of the spring and extending to 30 m radial distance into the salt flat (Fig. 1B). Soil samples (~100 g each) were taken from the surface (<10 cm depth) at 10-m intervals along each transect for a total of four samples per transect. All samples were kept on ice after retrieval and frozen upon return from the site. A subset of samples (30 g each) was stored at 4°C to maintain biological activity for microcosm experiments.

### Trace gas microcosms

Ten grams of each sample were placed into 160 ml serum vials, sealed with butyl rubber stoppers, and flushed with ultra-zero grade air. A trace gas mix (50 ml) was added to the vials to supplement the headspace with typical atmospheric mixing ratios of trace gases (2 ppmv CH_4_, 500 ppbv H_2_, and 100 ppbv CO). Microcosms were set up in triplicate for each sample in addition to a control vial of autoclaved soil to account for abiotic gas exchanges. An empty “blank” vial was used to control for potential leaks in the septa.

The headspace gas compositions were monitored via gas chromatography (Supplemental Methods). Initial survey microcosms were measured after 1 week to identify soils that exhibited microbial gas consumption and/or production. Subsequent microcosms were repeated under the same conditions for transect PQ3 with more frequent sampling (2-h intervals) to calculate gas consumption rates. PQ3 soils were selected due to their comparatively slower gas consumption, permitting more timepoints before reaching the detection limit for accurate rate measurements. The headspace trace gas concentrations were increased for samples with more rapid gas uptake to ensure that the gas depletion could be monitored before dropping below detection limits. Gas uptake was quantified via first-order reaction rate laws using the data points that followed log-linear behavior. Net rates were converted to pmol per gram dry weight of soil using the gravimetric soil water content (SWC) of the soils in order to standardize for variable soil mass in the microcosms due to differing SWC.

To characterize the trace gas oxidation kinetics of the 20- and 30-m soils of PQ3, microcosms were repeated across a range of H_2_ and CO concentrations (0.5 to 60 ppmv). Michaelis–Menten kinetic parameters (apparent *K_M_* and *V*_max_) were estimated using a non-linear least-squares model. Gas concentrations for these metrics were converted to molar values using Henry’s Law with an approximated headspace pressure of 1.3 atm based on the pressurization of the serum vials during addition of the trace gas mix.

### DNA extraction and metagenomic sequencing

Total DNA was extracted from subsamples of each soil using the DNeasy PowerMax Soil Kit (Qiagen, Carlsbad, CA) following the manufacturer’s protocol with modifications for low biomass samples (Supplemental Methods). The extracted mass of the soils varied from 10 to 30 g to achieve appropriate concentrations for downstream sequencing (Table S1). DNA yields were quantified with a Qubit Fluorometer and dsDNA HS Assay Kit (Thermo Fisher Scientific, Waltham, MA), and DNA size distributions were determined via capillary electrophoresis with an Agilent 2100 Bioanalyzer (Agilent, Santa Clara, CA).

Libraries for the extracted DNA samples were prepared using the Nextera DNA Flex Library Prep kit (Illumina, San Diego, CA). A total of 12 libraries were pooled and sequenced (2 × 150 bp) on a shared lane of a NovaSeq SP Flow Cell using a NovaSeq 6000 System (Illumina) at the Genomics Core Facility, Princeton University. A total of ~590 million paired-end reads of 150 bp length were generated across all 12 metagenomes (Table S1).

### Metagenome processing, assembly, and binning

Quality-filtered metagenomic reads from each metagenome were assembled with SPAdes v3.14.1 using the metaSPAdes platform (k-mer lengths 21, 33, 55, 77) [47]. Coverage was determined by mapping reads back to the assembled contigs with Bowtie v2.4.1 [48] in “very-sensitive” mode. Contigs were binned using the metaWRAP [49] binning and bin refinement pipelines, choosing the best bins derived from the MetaBAT2 v2.12.1 [50], CONCOCT v1.0.0 [51], and MaxBin v2.2.6 [52] binning algorithms with a minimum contig size of 1500 bp. The quality of the bins was assessed based on completeness and contamination estimates from CheckM v1.1.2 [53]. Bins were classified as high-quality (completeness >90%, contamination <5%) and medium-quality (completeness >50%, contamination <10%) metagenome-assembled genomes (MAGs) or otherwise excluded from further analyses. Taxonomic assignment of the MAGs was achieved using the Genome Taxonomy Database Toolkit (GTDB-Tk v2.2.6) [54] against GTDB R207.

### Community diversity analysis

Taxonomic classifications for the entire metagenomes were performed on the processed reads with Kaiju v1.9.0 [55]. Reads were also grouped into operational taxonomic units (OTUs) clustered to the genus level using the SingleM pipeline v1.0.0beta5 for community-level diversity analyses and statistics (https://github.com/wwood/singlem). Ribosomal protein L16 (*rplP*) was selected as the marker gene for the calculation of diversity metrics due to its Good’s coverage and abundance in the samples. Beta-diversity among communities was assessed based on Bray–Curtis dissimilarity of rarefied read counts. Unconstrained (non-metric dimensional scaling; NMDS) and constrained (distance-based redundancy analysis) ordination analyses were performed to visualize community variation and assess its association with environmental parameters. All ecological and statistical analyses were performed in R v4.2.0 with the vegan package v2.6-4 [56, 57].

### Metabolic characterization

Open reading frames on all contigs were predicted via Prodigal v2.6.3 [58]. The identified functional genes were annotated with a suite of Hidden Markov Models (HMMs) provided by Anvi’o [59], the National Center for Biotechnology Information Clusters of Orthologous Genes (NCBI COG) database [60], and the Kyoto Encyclopedia of Genes and Genomes (KEGG) database using the KofamKOALA HMMs [61]. Marker genes for major metabolic pathways were retrieved from the Kofam annotations to quantify the relative abundance of specific metabolisms (Supplemental Methods). Gene abundances were compared based on the number of mapped reads per gene length per total mapped reads in the metagenome (i.e. reads per kilobase per million mapped reads; RPKM), normalized to the average RPKM values of single-copy ribosomal genes (Supplemental Methods). The presence of specific metabolisms within MAGs was predicted using methods adapted from previously published methods combining the presence of metabolism-specific marker genes with overall pathway completion [38]. Specifically, autotrophic potential was confirmed after identification of the marker genes encoding for the major proteins involved in each respective carbon fixation pathway (e.g. Calvin–Benson: RbcL, RbcS, and PrkB; reductive tricarboxylic acid: AclA/AclB or CcsA/CcsB/Ccl; Wood–Ljungdahl: Acs or Cdh) in addition to a total pathway completion of at least 60%. [NiFe]-hydrogenases and CO dehydrogenases (CODH; CoxL) were specifically targeted for homology-based searches via blastp [62] against manually curated databases based on those previously compiled and publicly available for classification by subgroups and species source to determine distributional differences in protein forms and their associated functions [63]. Maximum-likelihood trees for protein sequences classified as Group 1h [NiFe] hydrogenases (HhyL) and Form I CODHs (CoxL) were constructed via RAxML-NG v1.0.2 [64] (Supplemental Methods).

## Results

### Polloquere establishes a gradient of environmental and geochemical conditions with radial distance from the hot spring

**Figure 2:**
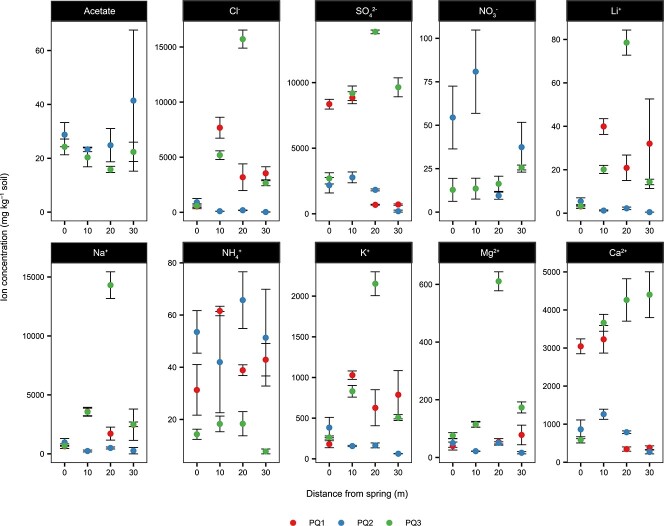
Ionic composition of the Polloquere soils; concentrations (mg kg^−1^ soil) of major ion species across radial distance from the spring in each transect; error bars represent ±1 standard deviation from the mean value of triplicate measurements; values below the detection limit are not shown (Table S2).

To provide context for the local environment of Polloquere, we measured the ionic composition of the spring water and surrounding soils (Fig. 2; Table S2). The spring contained high concentrations of chloride and sulfate (Cl^−^ and SO_4_^2−^; 750 ± 300 mg L^−1^ and 850 ± 15 mg L^−1^, respectively), which is characteristic of the athalassohaline Salar de Surire and the sulfur-rich volcanic system [42, 46] (Fig. S1). These ratios of Cl^−^ vs SO_4_^2−^ when compared to other springs in the ACVZ indicate that the spring is vapor-influenced with secondary water–rock interactions (Fig. S1). Previously published measurements of dissolved gas concentrations of other Surire springs in the region support this observation, revealing elevated levels of reduced gas species including H_2_, H_2_S, and CH_4_ [46]. Based on our measurements of gas compositions at the margin of the spring, concentrations of H_2_ ranged up to three times greater than typical atmospheric concentrations (maximum of 1500 ppbv). CO concentrations were orders of magnitude larger than background atmosphere, reaching a maximum of 1400 ppbv. These elevated quantities show evidence of gas emission from the spring. CH_4_ did not show significant enrichment, measuring within the bounds of typical atmospheric concentrations around 2 ppmv.

Anions extracted from the pore water of the surrounding soils displayed concentrations comparable to the Atacama Desert [65]. The highest SO_4_^2−^ concentrations were within range of the hyper-arid Yungay soils (~14 000 mg kg^−1^) and some Cl^−^ values exceeded Atacama concentrations (>3500 mg kg^−1^). Porewater Cl^−^ and SO_4_^2−^ concentrations ranged up to 3× and 20× those of average seawater, respectively. The organic carbon content of the soils (≤ 1.2%) was also typical of desert environments (Table S3). Ratios of soil C:N, primarily driven by a rise in organic carbon content, increased at greater distances from the hot spring across all three transects and resembled values from the Atacama and other Andean salt flats [66, 67].

The soil transects exhibited moisture and pH gradients to varying degrees as a function of increased distance from the spring (Table S2). All three transects followed the general trend of decreased gravimetric SWC from 0 to 30 m distance. PQ3 soils contained the highest average SWC, likely due to its downwind positioning relative to the spring. The sharpest drop in SWC also belonged to PQ3, declining by 27% from 0 to 10 m. The driest soils were within PQ2, where the most distal soil matched non-geothermal salt flat values that reached as low as 7%. In contrast to the spring water, most soils surrounding the spring were acidic with pH values ranging from 2.57 ± 0.05 to 5.18 ± 0.03 (Table S2). The 20- and 30-m soils from PQ3 were exceptions, approaching alkaline pH values more similar to the spring (pH = 8.81 ± 0.08).

### Polloquere establishes discrete biological zones with starkly different biomass loads and community compositions

Using sufficiently large differences in DNA recovery as a coarse indicator of biomass, extracted DNA concentrations from the soils suggest highly contrasting levels of biomass both among and within the transects (Table S1). PQ3 had relatively high DNA recovery adjacent to the spring (710 ng g^−1^), a severe drop to <10 ng g^−1^ at 10 m distance (8.78 ng g^−1^), and an eventual endpoint at ~2 ng g^−1^ in the distal soils. Similarly, PQ2 soils experienced a large decline in DNA between the 0- and 10-m soils, decreasing from ~100 ng g^−1^ to the lowest measured concentration among the soils at 0.65 ng g^−1^. In contrast, the PQ1 transect had low DNA recovery with less than 6 ng g^−1^ DNA in all PQ1 soils. Overall, most soil DNA extracts fell within range of the hyperarid, oligotrophic soils from the Yungay region of the Atacama Desert [68, 69]. The highest DNA concentrations occurred closest to the spring (PQ2-0m and PQ3-0m) and in the root-bearing sample nearest to desert plant life (PQ2-30m). Irregularities in the size distributions of the extracted DNA across distance also correlated with a decline in DNA quality in the more distal soils (Fig. S2).

**Figure 3:**
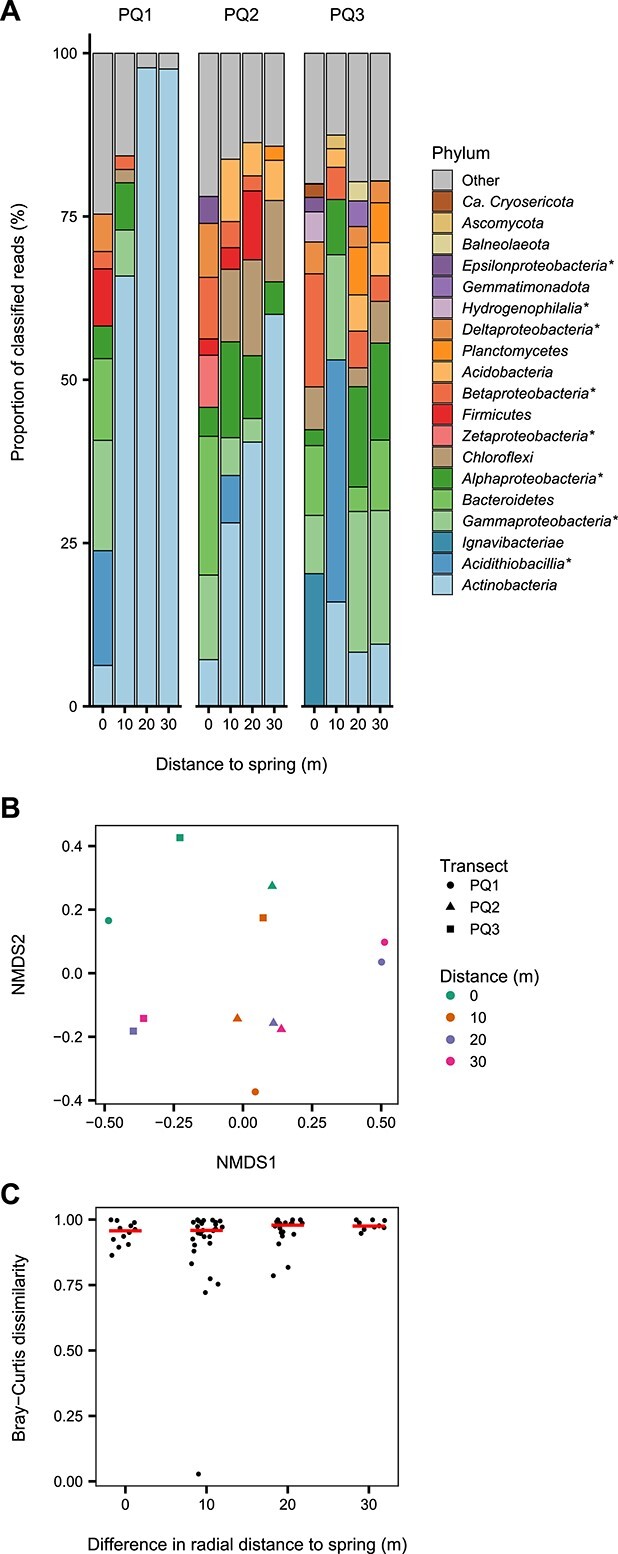
Community composition and diversity comparison of the soil microbial communities across the Polloquere transects; (A) taxonomic distributions of the soil microbial communities at the phylum level; quality-filtered reads were classified by comparison to the kaiju reference database; phyla representing ≥2% of the classified reads are shown; *Proteobacteria* are split into class-level assignments (marked with an asterisk) for greater resolution; (B) NMDS ordination of the pairwise distances (Bray–Curtis dissimilarity) between each community; (C) beta-diversity (Bray–Curtis dissimilarity) between sample pairs compared against the difference in sample radial distance to the Polloquere hot spring; all samples were compared both within and across the three transects; individual points are scattered for each group of distance differences to prevent obfuscation; horizontal lines represent the mean dissimilarity value for each difference in radial distance.

Along with the DNA concentrations and quality, the community compositions also varied both across and among the three transects. The samples exhibited high beta diversity with >75% dissimilarity between most soil communities at the genus level (Fig. 3C). The community variation did not follow a typical distance–decay relationship based strictly on spatial separation. Across all samples, only 20- and 30-m soils within each transect appeared to have more similar community structures relative to other samples in pairwise comparisons (Fig. 3B). Presence/absence of specific taxa (e.g. niches of thermophilic, halophilic, and acidophilic organisms) highlights the stark differences in composition among the more diverse soils (Fig. 3A and Fig. S3; Supplemental Results). Constrained ordination analysis suggests that neither the individual nor aggregated ion concentrations significantly correlated with the community structure. Of the remaining parameters, only pH (*P* = .007), SWC (*P* = .007), and spring distance (*P* = .02) explained proportions of the variance in the microbial communities with significance (Fig. S4). Combined, these constraining variables accounted for 38.8% of the total explained dissimilarity with an adjusted *R*^2^ of 0.16.

### Microbially mediated uptake of atmospheric H_2_ and CO increases across radial distance from Polloquere hot spring with varying affinities

**Figure 4:**
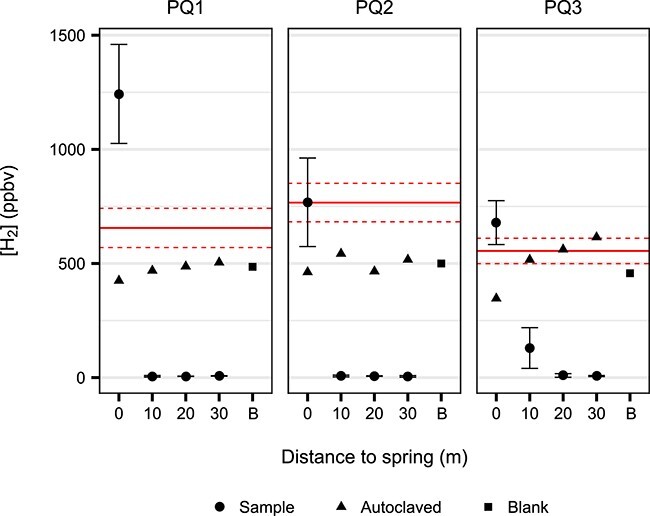
Atmospheric H_2_ depletion by soils in microcosms after 1 week; concentrations of headspace H_2_ after 1 week of soil microcosms supplied with an initial headspace of an ambient air gas mix; initial H_2_ concentrations are represented by the horizontal lines (solid line: mean; dashed lines = ±1 standard deviation); sample data points (circles) represent the mean of triplicate experiments with one standard deviation of error; autoclaved samples (triangles) and blank vials (squares; “B”) serve as controls for biological activity and vial leakage, respectively.

In transects PQ1 and PQ2, microcosms for soils from 10 to 30 m displayed complete consumption of atmospheric levels of H_2_ below detectable limits after 1 week (Fig. 4). PQ3 samples showed similar activity, with the 10-m soil exhibiting partial H_2_ depletion down to a ~100 ppbv threshold. Total consumption of CO was also observed in select distal soils from PQ2 and PQ3 (Fig. S5A). Aside from slight increases with considerable error, no significant change in CH_4_ concentrations was detected within the experimental duration or after extended timepoint measurements with elevated gas concentrations (Fig. S5B).

**Figure 5:**
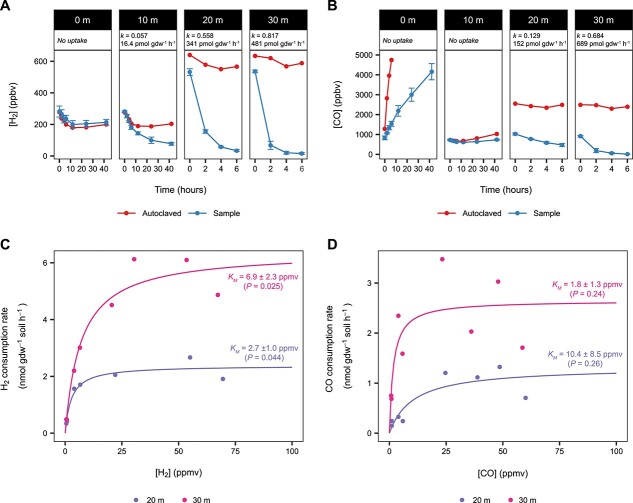
Rates and apparent kinetic parameters of microbial trace gas uptake in the Polloquere soils; (A, B) microcosm experiments displaying increased rates of atmospheric H_2_ (A) and CO (B) oxidation across radial distance from the Polloquere hot spring in soils from transect PQ3; sample data points represent the mean of triplicate experiments with one standard deviation of error; first-order reaction rates (per gram dry weight of soil; gdw) and constants (*k*) are listed for those soils that demonstrated gas depletion relative to the autoclaved soil controls; (C, D) nonlinear least-squares regression of the Michaelis–Menten kinetic model for H_2_ (C) and CO (D) oxidation in the 20- and 30-m soils from transect PQ3; apparent Michaelis affinity constants (*K_M_*) are shown with associated *P* values.

Shorter duration microcosms for PQ3 soils revealed the observed gas uptake to be rapid (Fig. 5A and B). For both H_2_ and CO, the quantified uptake rates are comparable to high-affinity gas uptake in lower-altitude desert regions [7, 21, 70]. However, H_2_ and CO uptake rates were not constant among the soils that exhibited gas depletion. The derived first-order oxidation rates for H_2_ and CO increased with radial distance from the spring. The largest incremental rate increase occurred between 10 and 20 m, where H_2_ consumption rates rose sharply from 16.4 pmol gdw^−1^ h^−1^ to 341 pmol gdw^−1^ h^−1^, corresponding to a ~10× increase in the rate constant. H_2_ consumption rates reached a maximum of 483 pmol gdw^−1^ h^−1^ (*k* = 0.817) at 30 m. Though CO concentrations remained stable at 10 m, a similar increase in CO uptake rates was observed between 20 and 30 m, rising from 152 pmol gdw^−1^ h^−1^ (*k* = 0.129) to 689 pmol gdw^−1^ h^−1^ (*k* = 0.684). The 0-m soil in contact with the spring water exhibited no detectable H_2_ change but increased in CO headspace concentration. CO emission also occurred in the heat-killed control microcosm at even higher rates than the biological replicates, which can be partially attributed to well-documented autoclaving effects [71, 72]. Additional targeted studies are required to deduce whether a natural chemical CO release mechanism is active in these soils.

To determine whether substrate affinity for the trace gases was also variable, we compared community-inferred estimates of Michaelis–Menten kinetic parameters between the 20- and 30-m soils for both H_2_ and CO (Fig. 5C and D). The apparent Michaelis constant *K*_*M*_ for H_2_ oxidation increased with distance from 2.7 ± 1.0 ppmv (2.7 ± 1.0 nM) to 6.9 ± 2.3 ppmv (7.0 ± 2.4 nM) of headspace gas. The shift indicates a slightly lower substrate affinity for hydrogenotrophic organisms at 30 m. Similar to the atmospheric uptake rates, the maximum estimated consumption rate of the 30-m soil (*V*_max_ = 6.4 nmol gdw^−1^ h^−1^) was ~2.5× larger than the 20-m soil. Together, the kinetic parameters suggest either a change in the abundance/activity of H_2_-oxidizing bacteria or a difference in the specific H_2_-oxidizing taxa present in the two communities. The apparent *K*_*M*_ of both soils can be defined as “high-affinity” (*K*_*M*_ < 100–150 nM) relative to other atmospheric H_2_-oxidizing bacteria/soils [15, 25, 73, 74]. CO showed opposite trends to H_2_ with respect to distance; however, the kinetic estimates lack the statistical significance to distinguish between the two soils confidently.

### Trace gas-scavenging is among the most prevalent metabolisms in the distal soils surrounding Polloquere

**Figure 6:**
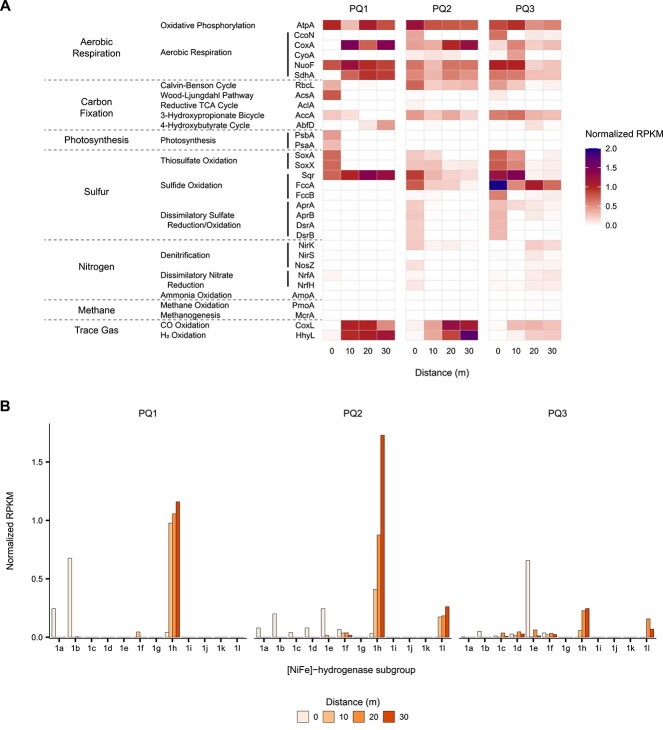
Shifts in the representation of major metabolic pathways among the soil metagenomes; (A) heatmap displaying the abundance of marker genes encoding for the major proteins involved in aerobic respiration, carbon fixation, photosynthesis, and metabolisms of sulfur, nitrogen, methane, and trace gases (H_2_, CO) in the soil metagenomes; (B) distribution of the subgroup (1a-l) classifications of group 1 [NiFe]-hydrogenases within the soil metagenomes; gene abundances are derived from metagenomic short reads and are reported as RPKM, normalized to the mean abundance of single-copy ribosomal marker genes.

Gene abundances from metagenomic analyses support the trends in gas uptake observed across the Polloquere soil transects and provide additional evidence for a complete shift in metabolic strategies. The relative abundances of marker genes for major energy and carbon pathways show an increased prevalence of trace gas-oxidizing metabolisms relative to other inorganic substrates in the most distal soils (Fig. 6A). From 0 to 10 m distance, sulfur metabolisms appear to be dominant with genes for the oxidation/reduction of multiple sulfur species present in the highest abundances. Beginning at the 10-m soils, genes for atmospheric H_2_ uptake (*hhyL*) and CO uptake (*coxL*) became more widespread in the metagenomes. In the case of H_2_, *hhyL* continued to increase incrementally with radial distance, whereas most sulfur-related genes declined in abundance to equivalent or lower levels compared to the trace gas metabolisms. Genes involved in photosynthesis were absent from all soils except PQ1-0m.

For a more thorough understanding of the H_2_-oxidation potential in the soils, we searched for all subgroups of the Group 1 [NiFe]-hydrogenases due to their role in H_2_ uptake as well as their phylogenetic and functional diversity (i.e. subgroups a–l). Hydrogenases were widely present in all soils (Fig. S6). The relative abundances of the various subgroups of Group 1 [NiFe]-hydrogenases revealed distinct profiles for each soil (Fig. 6B). The spring-adjacent soils contained sequences from subgroups a–f. Functionally, the most abundant groups at this distance for PQ2 and PQ3, 1b and 1e, are commonly found in bacteria with sulfur metabolisms [75]. Metabolic characterization of the MAGs derived from these soils verified this association, showing most high-quality MAGs that contain either of these hydrogenase subgroups also possess the potential to utilize sulfur species as well as some steps of denitrification (Table S5). Specifically, sulfur-oxidizing MAGs belonging to *Sulfurimonas*, *Sulfuricurvum*, and *Thiobacillus* were among the most prevalent MAGs present in these soils (Table S4). PQ1-0m and PQ2-0m also contained relatively elevated quantities of Group 1a, a hydrogenase widely characterized for its involvement in sulfate reduction. However, only one MAG containing this subgroup was recovered from PQ2-0m (Table S5). Given that this MAG did not exhibit the capacity for any sulfur metabolisms, the specific pathways associated with the Group 1a hydrogenases in the Polloquere soils cannot be confirmed. In the transitional 10-m soils, relative abundances of these three subgroups plummeted. Most hydrogenase sequences in these soils belong to subgroups involved in aerobic hydrogenotrophic respiration. In particular, Group 1h hydrogenases were the most abundant and are characterized by their ability to perform atmospheric H_2_ scavenging [75, 76]. In the distal 20- and 30-m soils, the vast majority of Group 1 [NiFe]-hydrogenases also belonged to this 1h subgroup, making up between 40% and 100% of all Group 1 hydrogenases in these soils. A more recently characterized hydrogenase, subgroup 1l, also appeared as the second most abundant Group 1 hydrogenase in the distal soils of PQ2 and PQ3. This group has also been found to mediate high-affinity H_2_ oxidation and is particularly common in Antarctic desert soils [13, 14, 20]. The increased abundance of 1h and 1l hydrogenases with increasing distance corroborates the observed increase in atmospheric H_2_ uptake by these soils. Subgroups 1f and 2a have also been shown to uptake atmospheric H_2_, but were either not detected (2a) or present at much lower relative quantities (1f) [77, 78].

**Figure 7:**
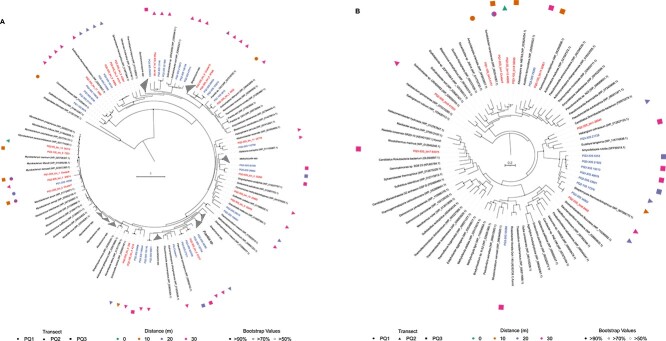
Phylogenetic analysis of group 1h [NiFe]-hydrogenases and form I CODHs derived from Polloquere soils; maximum-likelihood trees constructed using amino acid sequences of (A) the large subunit (HhyL) of group 1h [NiFe]-hydrogenases and (B) the large subunit (CoxL) of form I CODHs; the trees were bootstrapped with 1000 replicates and rooted with an outgroup of (A) group 1g [NiFe]-hydrogenase sequences and (B) form II CODHs (not shown); the tree scales are shown in the center of each tree and correspond to units of substitution per amino acid position; sequences derived from Polloquere soil metagenomes are in blue, with binned sequences belonging to high-quality (>90% completion, <5% contamination) and medium-quality (>50% completion, <10% contamination) MAGs highlighted in red; shapes surrounding the trees denote the samples from which the Polloquere sequences were recovered.

The phylogenetic diversity of the hydrogenases recovered from the transects provides evidence for variation in the H_2_-oxidizing bacteria inhabiting the soils (Fig. 7A and Table S4). The identified hydrogenases span many major bacterial phyla, including *Actinobacteriota*, *Proteobacteria*, and *Chloroflexota* members. Sequences derived from a variety of *Actinobacteriota* were the most prevalent, particularly from the 20- and 30-m samples from transects PQ2 and PQ3. These comprise typical soil bacteria groups such as *Acidimicrobiia* and *Actinomycetia*. A cluster of *Mycobacteria* sequences was also recovered with representatives from all transects and distances, including the sole high-affinity hydrogenase from a spring-adjacent soil MAG (PQ2-0m bin 15). Another group of divergent sequences belongs to a single MAG from PQ2-20m (bin 17) related to an undefined member of *Chloroflexota* (UBA4733).

Of the 22 recovered MAGs found to contain Group 1h hydrogenases, 15 were also shown to possess CODH enzymes, indicating a potential for CO oxidation (Table S5). Some of the CODH sequences derived from the *Mycobacteria* MAGs also share a clade related to other *Actinomycetia* sequences, and a clade of *Streptomyces* CODHs was recovered from unbinned contigs (Fig. 7B). The overlap in these genes within our MAGs may suggest that some organisms in the Polloquere soils are capable of oxidizing both H_2_ and CO, contributing to the observed uptake of both gases. The majority of the MAGs also contained partially complete carbon fixation pathways (primarily the Calvin–Benson cycle), suggesting they may have the capacity for autotrophy (Table S5).

## Discussion

This study demonstrates that the Polloquere hot spring exerts influence on its surrounding soils, altering their geochemistry and overall biology to produce distinct environments defined by their geometric arrangement relative to the hot spring. With increased radial distance from Polloquere, the influence of the spring diminishes, and the soil properties shift to conform with the more localized settings within the salt flat. The resulting effects are variable among the three transects as different geochemical properties diverge. However, general patterns can be observed in the inferred biomass load across distance. Though biomass cannot be directly inferred from the DNA concentrations due to external mineralogical and chemical effects potentially inhibiting DNA recovery, the observed macroscale trends in DNA abundance suggest large biomass variations among the soils despite the inability to characterize the absolute magnitude of these differences. In the case of transects PQ2 and PQ3, an apparent intensification of biological stressors to the microbial communities is reflected in the steep decline in DNA recovery. PQ1 DNA concentrations were comparable to the leanest soils of the other transects, though the apparent enrichment of DNA in the spring-adjacent soils did not persist. The cause of this discrepancy is not entirely evident, but less direct input of the spring water into the PQ1-0m soil could be a factor leading to lower biomass contribution from the spring. Environmental extremes, including aridity, large diurnal temperature fluctuations, and UV flux, are all regional characteristics of this salt flat that are likely to contribute to the less hospitable conditions of the soils that cause the apparent decline in biomass. Despite most of the soils exhibiting similar acidity independent of radial distance from the spring, increased pH values in the distal soils of PQ3 also explained a fraction of the community-level variance. Further attention to the roles of pH and other geochemical parameters as second-order effects is warranted in follow-up studies.

Within the soil microbial communities, we observed a consistent shift in the overall composition and metabolic landscape across radial distance in each transect. We show that energy metabolism preference changes from a primarily sulfur-driven system to one fueled by atmospheric H_2_ and CO scavenging as the distance from the spring increases. Though the lack of CH_4_ oxidation was somewhat unexpected given the abundance of CH_4_ in other Surire springs [46], high-affinity methanotrophy has been less frequently observed in soils with other trace gas uptake activity, unlike the common cooccurrence of H_2_ and CO oxidation [7]. Given that these trace gas metabolisms are fully aerobic processes, the coinciding depletion of O_2_ (e.g. via respiration) could also have played a role in the varied consumption rates both among and within the microcosm experiments. However, such effects would be minor as H_2_, CO, and CH_4_ are assumed to be the limiting reactants following first-order reaction rate laws compared to the higher concentrations of O_2_ by orders of magnitude. The persistent consumption of the trace gases throughout the duration of the microcosms suggests that functional anoxia was never induced as this would completely shut down the oxidation activity rather than the expected slowdown as observed while the trace gas concentrations declined.

The experimental and metagenomic evidence of biological oxidation of H_2_ and CO in the Polloquere soils complements the findings from similar high-altitude desert environments, suggesting that the utilization of trace gases enables microbial persistence under environmental stress. The Polloquere soils highlight this role of trace gas metabolisms in bacterial survival by comparing soil microbial communities across a short spatial scale and environmental gradient with decreasingly hospitable conditions away from the hot spring. Within the span of 30 m, high-affinity hydrogenotrophs and carboxydovores transition from absent/inactive to abundant, highly active bacteria as the soils become increasingly dry and more directly exposed to the local ambient conditions of the salt flat.

The prevalence of organisms capable of oxidizing both H_2_ and CO based on the co-occurrence of *hhyL* and *coxL* genes in most of the MAGs containing Group 1h hydrogenases suggests that the observed atmospheric uptake of both gases may be partially performed by individual organisms rather than independent taxa. The presence of both uptake hydrogenases and CODHs has been observed in other MAGs derived from different soil environments [8, 11], and the dual ability for H_2_ and CO oxidation has recently been demonstrated in cultured *Chloroflexota* isolates in response to nutrient limitation [24, 26]. Select *Mycobacteria*, including relatives to Polloquere H_2_-oxidizing MAGs such as *Mycobacterium smegmatis*, have also been shown to utilize both H_2_ and CO at concentrations <10 and <50 ppmv, respectively, though the extent of their affinity for atmospheric concentrations is not explicitly described [79]. Additionally, a MAG from PQ2-30m (bin 11) classified as a *Ktedonobacteria* species contains a hydrogenase closely related to *Ktedonobacter racemifer*, which has previously been isolated from volcanic soils and shown to take up atmospheric CO along with other *Ktedonobacteria* member *Thermogemmatispora* sp. T81 also oxidizing H_2_ [26, 30, 80–82]. The ability of mixotrophic bacteria to oxidize both gases could serve as an additional advantage in oligotrophic soils by enhancing metabolic flexibility.

The metagenomic evidence highlights H_2_ as an important substrate across the entirety of the soil transect. The abundance of Group 1 [NiFe]-hydrogenases responsible for H_2_ uptake remained consistently high in all soils. Proximity to Polloquere mainly affected the rates, kinetics, and metabolic roles of the H_2_ uptake. Though no consumption was observed at 0 m, the most abundant hydrogenases in these soils are present within sulfur-oxidizing organisms. These enzymes have a lower affinity for H_2_ compared to the Group 1h or 1l hydrogenases and are likely able to function *in situ* due to the elevated concentrations of H_2_ emitted by Polloquere or delivered through the subsurface from the geothermal source water. As the soils become more separated from the spring, rates of H_2_ uptake rise by a factor of 30 in the PQ3 microcosms. In addition, the apparent H_2_ affinities (*K*_*M*_) of the 20- and 30-m soils fall within the bounds of high-affinity hydrogenase uptake activity observed in other soils exhibiting atmospheric uptake [15, 73]. The *K*_*M*_ values calculated in this study (< 10 nM H_2_) are the lowest values reported to date as measured from whole soils or cultured organisms, indicating a particularly strong affinity for H_2_ substrate [73, 74].

The changes in H_2_ oxidation rate and affinity cannot be attributed to an increased absolute abundance of trace gas-oxidizing bacteria. Using the extracted DNA quantities and the relative abundances of *hhyL* to roughly estimate the number of high-affinity H_2_-oxidizing bacteria, we approximate that there are more trace H_2_-scavenging organisms per gram of soil at 10 m than there are in either the 20- or 30-m soils despite the opposite trend in atmospheric H_2_ uptake rates. Therefore, we conclude that the changes in rates and affinities are primarily controlled by the individual enzyme kinetics and metabolic preferences of the specific H_2_ oxidizers present in each soil. Indeed, the recovered MAGs containing Group 1h hydrogenases are phylogenetically varied among the soils, displaying taxonomic diversity in H_2_ oxidizers. The activities of these trace gas oxidizers may also change depending on the soil environment. Given that trace gas scavenging is often a facultative pathway employed under stress, these metabolisms may be preferentially upregulated and/or activated in bacteria at the greater distances where harsh conditions stimulate their utilization.

Based on the multi-factor gradients observed across the Polloquere soil transects and the high diversity among the soil microbial communities, we outline a model characterizing the environments surrounding the hot spring consisting of three spatial zones: spring-adjacent, transitional, and distal. Each zone is defined by a unique set of environmental conditions and microbial communities dependent on the proximity of the soils to Polloquere hot spring. The concept expands upon the “islands of biodiversity” created by fumarolic soils as previously described in an Andean system near Socompa Volcano [41]. Although past studies of Andean soil environments supported by local geothermal energy have focused on the increased diversity of the microbial communities compared to arid soils using 16S/18S rRNA gene surveys [41, 83, 84], this work provides detailed metabolic context to explicitly determine functional differences among the soil communities. Unlike other reports of fumarole-supported communities, we did not find evidence of any photoautotrophy. Primary productivity in the spring-adjacent soil appears to be mainly contributed by sulfur-oxidizing autotrophs with the Calvin–Benson cycle being the most represented carbon fixation pathway (Table S5). In the distal soils, trace gas oxidation may be among the most dominant sources of primary productivity akin to polar deserts [10, 11, 14] and other arid soils with limited photosynthetic activity [12, 85]. The presence of carbon fixation pathways in many of the hydrogenotrophic MAGs, specifically the Calvin-Benson pathway, suggests that the energy derived from trace gas oxidation may support both vital cell functions for survival as well as primary production for acquiring carbon in the distal Polloquere soils. The sampled Polloquere transects also enable a more extensive spatial comparison relative to the geothermal source to highlight gradients rather than binary differences. As a result, we captured the transition from the diverse communities of thermophilic and sulfur-dependent organisms in the spring-adjacent soils to the trace gas scavenging communities of the more salt flat-influenced distal soils coinciding with progressive changes in soil properties and biomass. This also enabled us to determine the extreme dissimilarity between communities both within and among each transect, which was a key factor in defining the 10-m soils as a separate “transitional” zone distinct from the geothermal and salt flat soils. The existence of a transitional zone as well as the presumptive effect of emitted gases on the apparent H_2_ and CO rates/affinities across the gradient suggests that the range of influence of Polloquere on the local microbiology extends beyond the immediate vicinity of the spring in the adjacent soils (0-m). Instead, Polloquere effectively serves as the center of the island, establishing a collection of micro-ecosystems that eventually conform to the regional environment.

Overall, this study illustrates the degree of influence the Polloquere hot spring exerts on the surrounding soils and their microbial communities, particularly in developing distinct metabolic niches as a function of radial distance. Polloquere and the Salar de Surire are added to a growing list of both arid and high-elevation environments that contain diverse trace gas scavenging bacteria as abundant members of their soil communities and represent the first instance of a gradient change in H_2_ and CO uptake rates and community compositions based on meter-scale spatial distribution in a geothermal setting. Further *in situ* measurements are required to improve understanding of the gas sources and fluxes across distance as well as how regular temporal cycles and variables within the environment affect the gas uptake rates. The transitional model across the radial distance proposed for Polloquere may represent a typical organization of similar geothermal environments in extreme oligotrophic settings. Analyses of additional systems within the Andean Altiplano and other high-elevation volcanic regions should be performed to confirm the commonality of the proposed gradient model.

## Supplementary Material

Garvin2024_Polloquere_Supplemental_wrae062

## Data Availability

All raw metagenomic sequences and metagenome-assembled genomes were deposited in the NCBI Sequence Read Archive and have been made publicly available under the BioProject PRJNA758125.
