# Supplementary material for: Prevalence of trace gas-oxidizing soil bacteria increases with radial distance from Polloquere hot spring within a high-elevation Andean cold desert
Source: ISME J. 2024 Apr 16;18(1):wrae062. doi: 10.1093/ismejo/wrae062 (PMC11094475; doi:10.1093/ismejo/wrae062)
Supplement: Garvin2024_Polloquere_Supplemental_wrae062 [file garvin2024_polloquere_supplemental_wrae062.pdf]

Supplementary Information for:

Prevalence of trace gas-oxidizing soil bacteria increases with radial distance from Polloquere hot spring within a high-elevation Andean cold desert

Zachary K. Garvin[1]*, Sebastian R. Abades[2], Nicole Trefault[2], Fernando D. Alfaro[2], Katie Sipes[3,4], Karen G. Lloyd[3], Tullis C. Onstott[1]**

[1]Department of Geosciences, Princeton University, Princeton, NJ, USA

[2]GEMA Center for Genomics, Ecology & Environment, Universidad Mayor, Camino La Pirámide, Santiago, Chile

[3]Department of Microbiology, University of Tennessee, Knoxville, Knoxville, TN, USA

[4]Department of Environmental Sciences, Aarhus University, Roskilde, Denmark

*Corresponding author: zgarvin@princeton.edu

**Deceased

This file includes:

Extended Methods

Extended Results and Discussion

Figures S1-S6

Tables S1-S5

References

MATERIALS AND METHODS

Analysis of soil and water ion species

To determine the ionic compositions of the soils, aqueous extractions were performed in triplicate for each sample to obtain the soluble species for ion chromatography. Frozen samples were first homogenized via mortar and pestle. The pulverized soils were placed in deionized water at a 1:5 w/v ratio and leached for 24 hours. The slurries were centrifuged at 2000 RPM for 20 minutes and supernatants were obtained. The aqueous extracts were analyzed with a Dionex ICS-5000+ Capillary HPIC system (Thermo Fisher Scientific, Waltham, MA, USA) to quantify major ionic species. Anions were separated using a Dionex IonPac AS15 analytical column (2 x 250 mm inner diameter, I.D.), Dionex IonPac AG15 guard column (2 x 50 mm I.D.) and Dionex ADRS 600 suppressor (2 mm; current = 52 mA) with an eluent of potassium hydroxide at a flow rate of 0.35 mL/min using a gradient from 1 mM to 60 mM for 70 min. Cations were separated using a Dionex IonPac CS16 analytical column (3 x 250 mm inner diameter, I.D.), Dionex IonPac AG15 guard column (3 x 50 mm I.D.) and Dionex CRDS 600 suppressor (2 mm; current = 29 mA) with an eluent of methanesulfonic acid at a flow rate of 0.35 mL/min and an isocratic elution of 28 mM for 35 min. Water samples were diluted and run directly using the same parameters.

Soil carbon and nitrogen measurements

Soil samples were finely ground with mortar and pestle, followed by lyophilization to prepare for C, N, $\delta^{13}C$, and $\delta^{15}N$ measurements. Soil water content was measured gravimetrically by water loss after lyophilization. The homogenized, freeze-dried soils were analyzed using a Costech ECS4010 Elemental Analyzer (Costech Analytical Technologies, Valencia, CA, USA) coupled to a Thermo-Finnigan Delta+XL mass spectrometer via a Thermo-Finnigan Conflo III device at the Stable Isotope Laboratory at the University of Tennessee, Knoxville, USA. The instrument was run with helium as the carrier gas and furnace temperatures set to 1050°C (oxidation) and

650°C (reduction). The freeze-dried soil was partitioned into two treatments to determine the organic and inorganic content of the soil. Inorganic carbon was volatized via acid treatment of the soil with 1 N HCl and subsequent oven drying at 45°C for 56 hours prior to measurement.

Trace gas microcosms

The headspace of each microcosm vial was over-pressurized to ~1.5 atm to maintain positive pressure and prevent atmospheric contamination. All vials were maintained at ambient room light and temperature conditions. The headspace gas compositions of the microcosm vials were measured via two gas chromatographs (Peak Laboratories, Mountain View, CA, USA) with separate detectors for quantification of specific gases (calibrated detection limits in parentheses): reducing compound photometer for H_2 (10 ppbv) and CO (10 ppbv), and flame ionization detector for CH_4 (50 ppbv). Survey microcosms were sampled after one week of incubation, whereas measurements for the rate-calculating microcosms of PQ3 were made at 2-hour intervals for the first 8 hours, followed by longer intervals until a total duration of 42 hours.

DNA extraction for low biomass soils

The DNeasy PowerMax Soil Kit (Qiagen, Carlsbad, CA) protocol was amended with two freeze-thaw steps after initial homogenization and lysis, as previously described [1]. Briefly, the homogenized soil suspended in the Qiagen "C1" lysis solution was incubated at 99°C for 2 min and subsequently frozen overnight at -20°C. The soil solutions were then thawed and re-frozen overnight prior to a repeated homogenization step for 10 min. Blank extractions were subjected to the same procedures using only reagents to control for contamination.

Metagenomic reads quality-filtering

The raw metagenomic reads were processed using TrimGalore [2] to trim low-quality 3' ends (Phred < 20) and to remove Illumina adapter sequences. Reads were then filtered for quality

using the FASTX toolkit (Phred ≥ 30) [3]. Reads derived from potential human contamination were removed using KneadData 0.7.7-alpha (https://github.com/biobakery/kneaddata).

Metabolic and single-copy gene targets

The assembled metagenomes were searched for metabolic marker genes from the KEGG database via KofamKOALA HMMs [4]. The targeted metabolisms and their associated KEGG KO identifiers include: aerobic respiration (K02111 *atpA*, K00404 *ccoN*, K02274 *coxA*, K02297 *cyoA*, K00335 *nuoF*, K00239 *sdhA*), carbon fixation (K01601 *rbcL/cbbL*, K00198 *acsA*, K15230 *aclA*, K01962 *accA*, K14534 *abfD*), photosynthesis (K02703 *psbA*, K02689 *psaA*), sulfur oxidation/reduction (K17222 *soxA*, K17223 *soxX*, K17218 *sqr*, K17230 *fccA*, K17229 *fccB*, K00394 *aprA*, K00395 *aprB*, K11180 *dsrA*, K11181 *dsrB*), nitrogen oxidation/reduction (K00368 *nirK*, K15864 *nirS*, K00376 *nosZ*, K03385 *nrfA*, K15876 *nrfH*, K10944 *amoA*), methanotrophy (K10944 *pmoA*), and methanogenesis (K00399 *mcrA*). Predicted proteins were also searched for hydrogenases and CODHs (CoxL) against manually-curated databases based on previously published and publicly available databases [5]. Search results were filtered for hits with E-value < 1e-05, bit score > 40, and coverage > 80%. Based on these criteria, the single best annotation was selected for each hit and used to classify Group 1 [NiFe]-hydrogenases into subgroups a-l and CODHs into Forms I and II of CoxL.

All gene abundances were normalized to the average RPKM of bacterial and archaeal single-copy ribosomal genes retrieved from Anvi'o HMM search: Ribosom_S12_S23, Ribosomal_L1, Ribosomal_L16, Ribosomal_L3, Ribosomal_L6, Ribosomal_S2, Ribosomal_S7, and Ribosomal_S9.

Phylogenetic analyses of trace gas-oxidizing MAGs

Amino acid sequences of Group 1h [NiFe]-hydrogenases with a minimum length of 300 a.a.'s were clustered with CD-HIT v4.8.1 to group identical sequences [6]. The filtered sequences

were aligned with reference sequences from the Group 1 [NiFe]-hydrogenases contained in the BLAST database using MAFFT v7.515 [7] with the L-INS-i method. ModelTest-NG v0.1.7 [8] was used to select the LG+I+G4 amino acid substitution model for phylogenetic analysis of the alignment. A maximum-likelihood phylogenetic tree was constructed via RAxML-NG v1.0.2 [9] with 1000 bootstraps to assess confidence in the topology. The tree was rooted with sequences classified as Group 1g as an outgroup.

A maximum-likelihood tree for Form I CODHs was also created using the same methodology. The amino acid sequences were filtered with a minimum length of 500 a.a.'s. The tree was constructed with the LG+G4 model and included Form II CODH sequences as an outgroup.

RESULTS AND DISCUSSION

Characterization of the Polloquere hot spring geochemistry

Comparison of Polloquere Cl^- and SO_4^{2-} concentrations with those of other hydrothermal systems in the Surire region and salars throughout northern Chile suggests that Polloquere can be grouped with a subset of Surire springs containing elevated SO_4^{2-}, which is indicative of H_2S dissolution and/or leaching (Fig. S1) [10]. These springs are defined as mixed or intermediate waters influenced by secondary gas-water-rock interactions as opposed to more mature waters sourced primarily from subsurface hydrothermal fluids [10, 11]. This classification is further supported by the moderately alkaline pH (8.81 ± 0.08), which excludes the spring from being a purely vapor-phase acid-sulfate system sourced from meteoric water.

The presence of specific taxa characterizes the change in soil community compositions

PQ1 soils had the smallest number of unique taxa representatives, shifting from diverse members of *Proteobacteria* to predominantly *Actinomycetia* across the radial distance from the spring (Fig. 3 and Fig. S3). Soils in transect PQ2 experienced a similar trend of increasing *Actinobacteria*

abundance in the more distal soils, but distinguished themselves by a higher relative proportion of other typical soil bacterial phyla such as *Firmicutes*, *Chloroflexi*, and *Acidobacteri*a. Among the *Chloroflex*i members, *Ktedonobacteria* were the most prevalent at 20 and 30 meters. Members of *Ktedonobacteria* have been found in other arid and geothermal environments, and isolates have been shown to oxidize both CO and H_2 [12–16]**.** PQ3 displayed the most influence from the spring adjacency with higher abundances of thermophilic organisms from 0-10 meters, such as *Ignavibacteriae* and *Candidatus Cryosericota* (likely relative of thermophile *Caldiserica*), reflecting the influence of the geothermal heat. The two most distal soils shared a general community structure reminiscent of other arid soil environments at the phylum level [17, 18]. PQ3-20m also featured a higher abundance of halophilic taxa (e.g., *Balneolia*), which is consistent with its salt concentrations being the highest of all samples (Table S2). Though the communities exhibited minimal overlap, a unique niche of acidophilic, sulfur-oxidizing *Acidithiobacillia* was supported in all three transects (PQ1-0m, PQ2-10m, and PQ3-10m). The soils are clearly defined by distinct microbial communities, which correlate with their distance from the spring and the concomitant environmental changes.

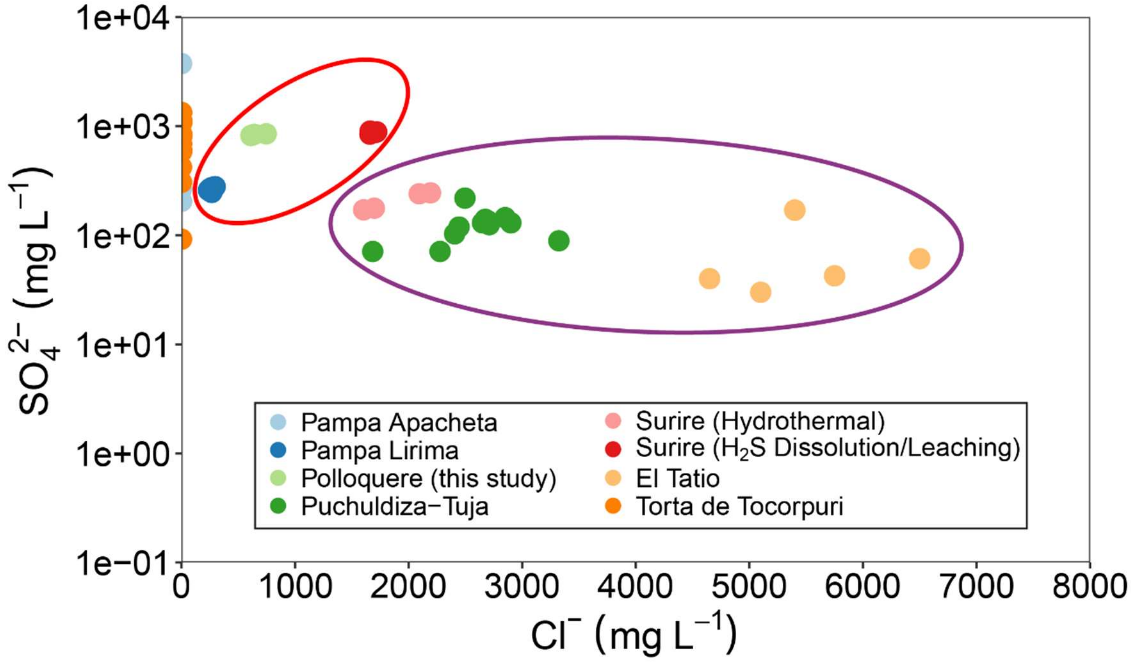

Fig. S1. SO_4^{2-} vs. Cl^- concentrations in the Polloquere hot spring and other hydrothermal systems within Andean salt flats of northern Chile. Additional salt flat data was retrieved from previously published measurements [10]. The red gate encompasses “intermediate” waters undergoing secondary gas-water-rock interactions, whereas the purple gate encompasses “mature” waters fed from the hydrothermal source water. Ungated sulfate-rich waters along the y-axis are “vapor-phase” springs sourced from steam-heated, shallow aquifers with significant meteoric water input.

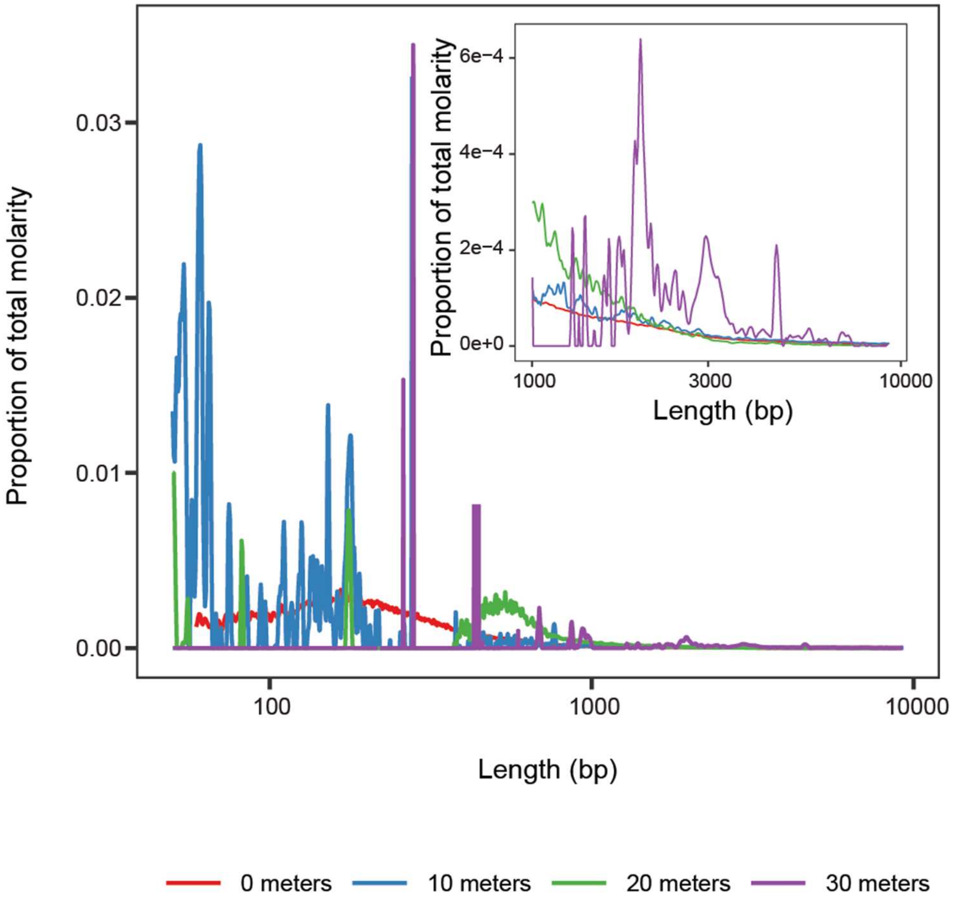

Fig. S2. Size distributions of the extracted DNA molecules from transect PQ3 in base pairs (bp). Values were normalized to the total molarity (pmol L^{-1}). The inset shows an enlarged view of the 10^3-10^4 bp region, which accounts for 15% (0 m), 24% (10 m), 20% (20 m), and 46% (30 m) of the total molarity for each sample.

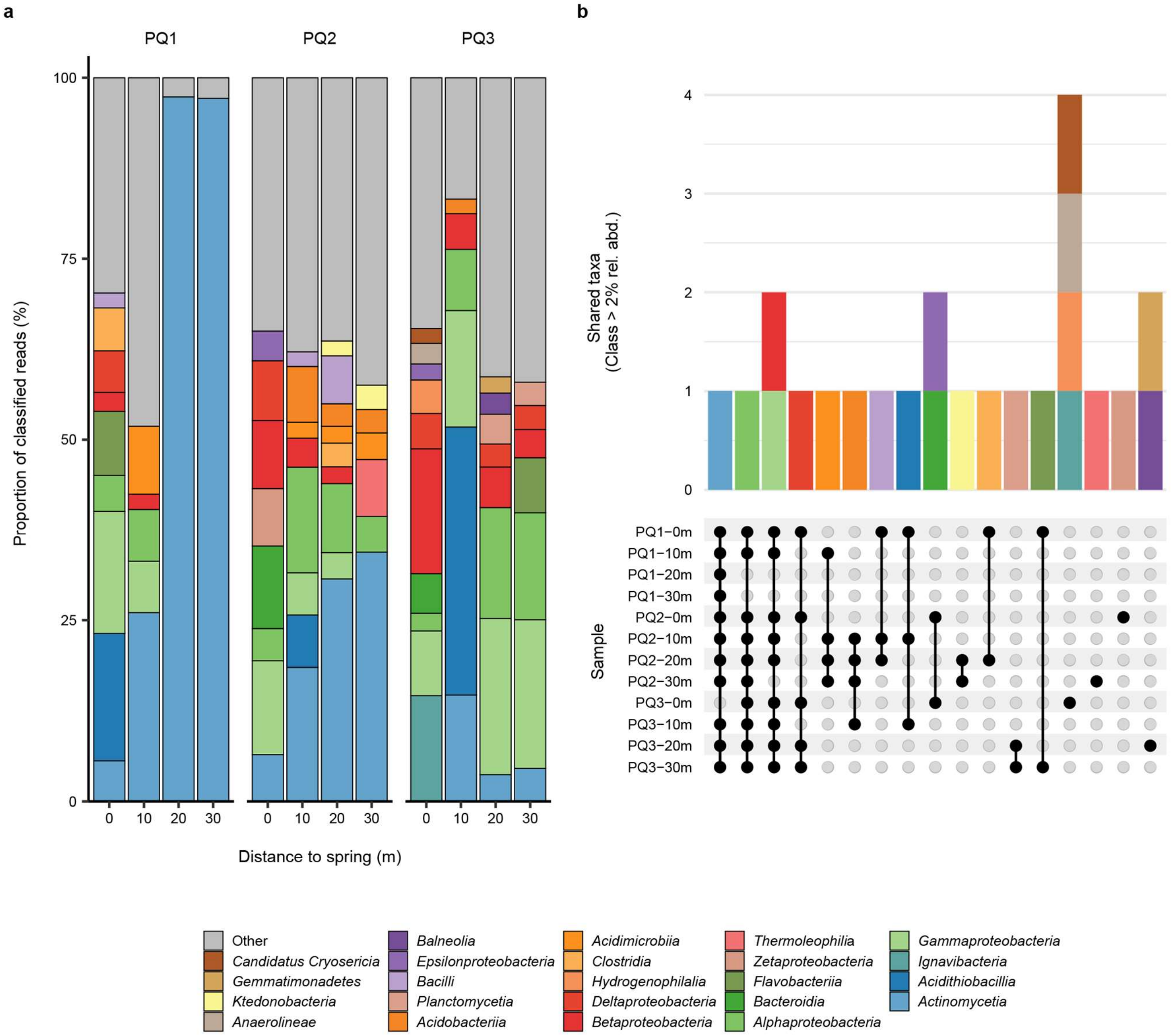

Fig. S3. Relative abundance (**a**) and presence/absence overlap (**b**) of taxa in the soil microbial communities across the Polloquere transect at the Class level. Quality-filtered reads were classified by comparison to the Kaiju reference database. Classes representing >2% of the classified reads are shown.

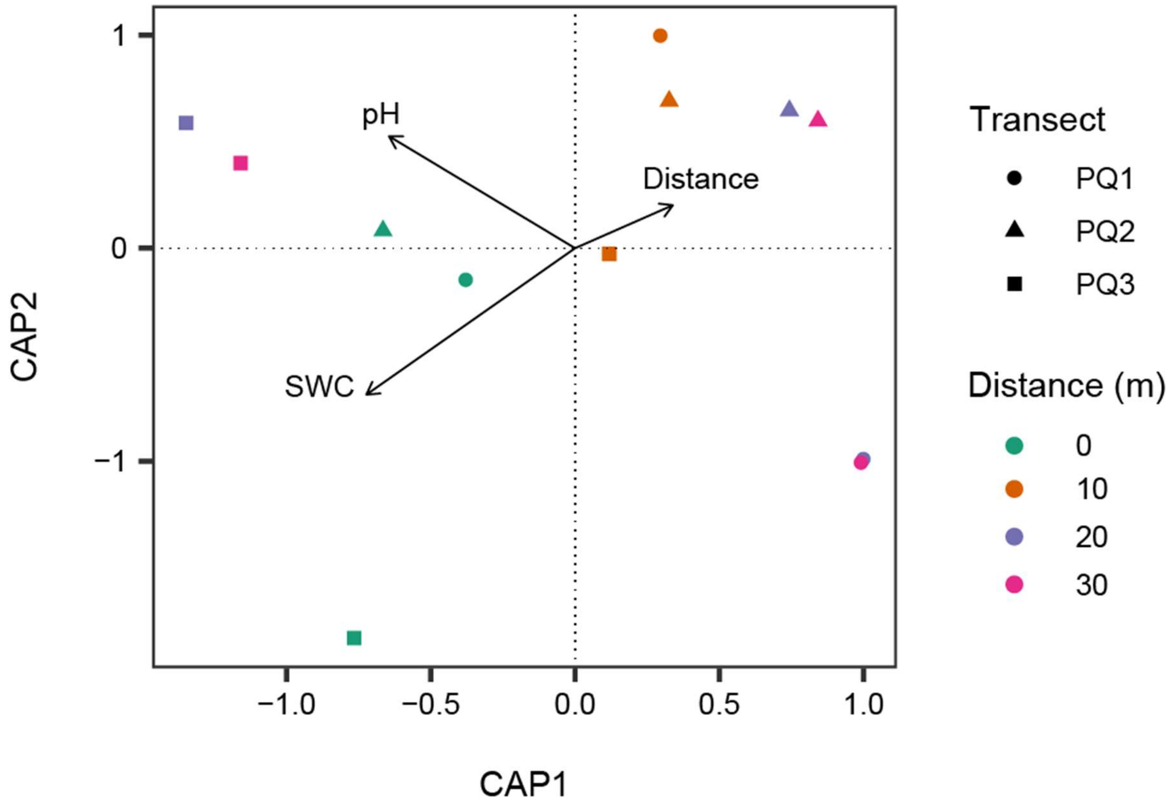

Fig. S4. Distance-based redundancy analysis (dbRDA) of pairwise distances (Bray-Curtis dissimilarity) between each community. Model selection was performed via forward stepwise selection with permutation tests for a maximized R^2 using geochemical and physicochemical parameters as potential explanatory variables. Significance of the constraining variables was evaluated via Monte Carlo permutation tests. SWC (P = 0.007), pH (P = 0.007), and spring distance (P = 0.02) were chosen for the model, accounting for 38.8% of the total community distance with an adjusted R^2 of 0.16.

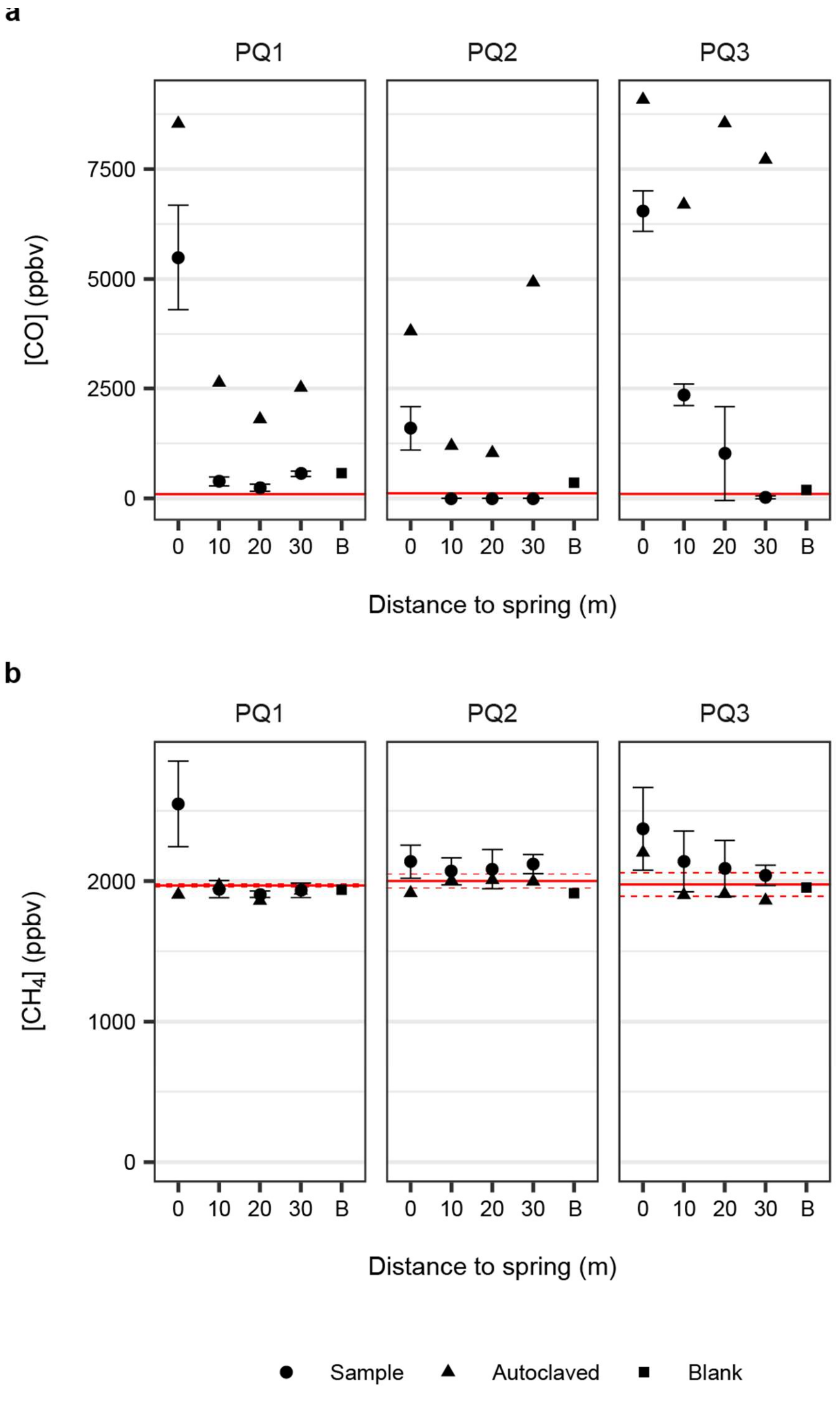

Fig. S5. Survey microcosm experiments displaying change in the headspace (**a**) CO and (**b**) CH_4 concentrations after 1 week. Red lines indicate the starting headspace concentrations of each gas (red line = mean value, dashed lines = 1 SD). Sample data points (circles) represent the mean of triplicate experiments with 1 SD of error. Biologically-induced changes were assessed relative to the autoclaved soil controls (triangles) and blank vials (squares).

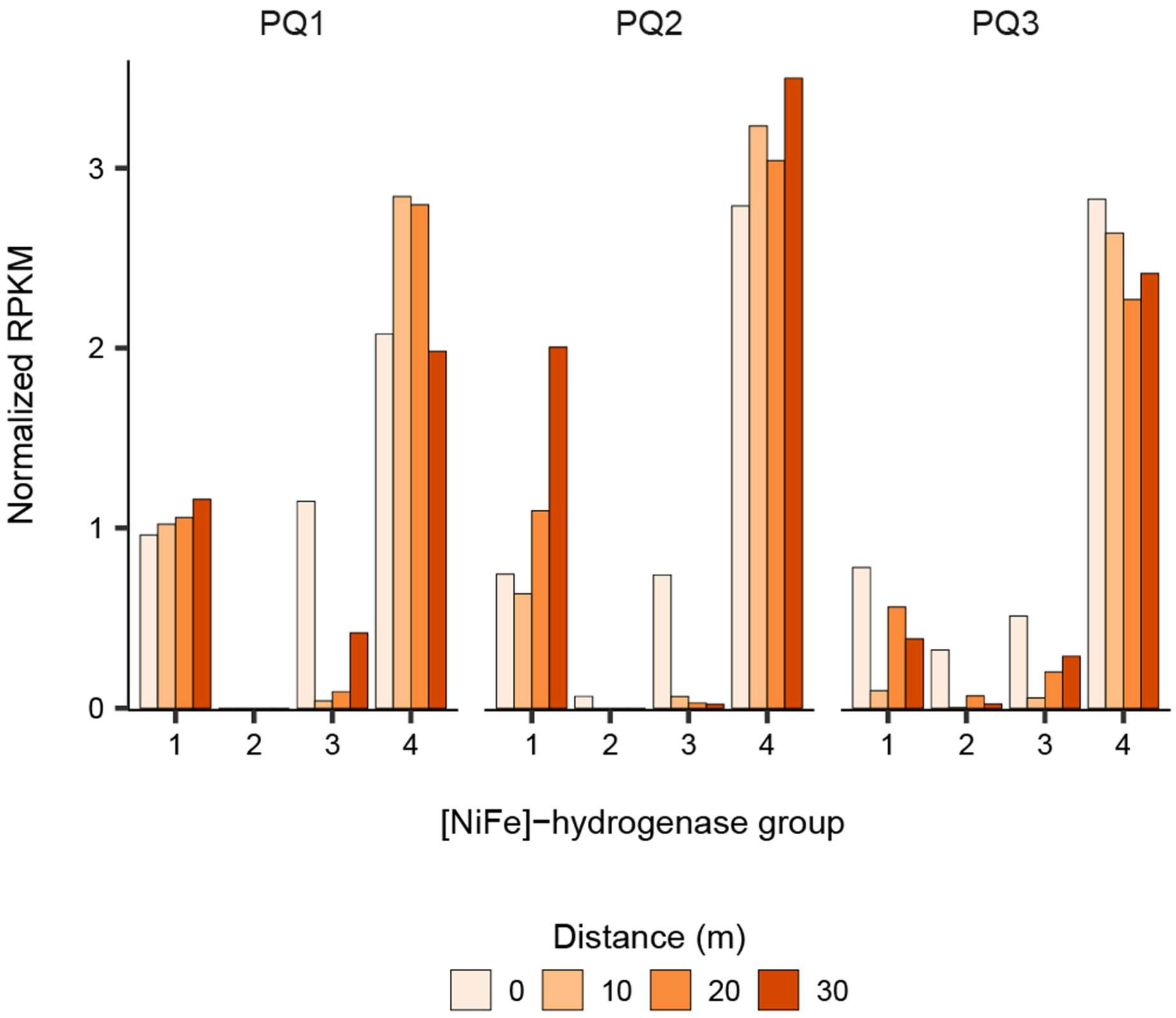

Fig. S6. Distribution of major group classifications (groups 1-4) for all [NiFe]-hydrogenases within the soil metagenomes.

Table S1. Metagenome quality control and processing statistics

Transect	Distance (m)	Soil extraction weight (g)	DNA concentration (ng g^{-1} soil)	Read Direction	Read Count				
					Raw	Trimmed	Filtered (Q=30)	Contamination Removal	Total
PQ1	0	30	0.71	Forward	6,965,508	4,912,166	3,468,024	3,091,856	6,344,701
				Reverse	6,965,508	4,984,442	3,861,553	3,252,845	
	10	30	1.01	Forward	3,124,656	3,029,625	1,694,296	1,620,548	4,007,357
				Reverse	3,124,656	3,028,490	2,588,402	2,386,809	
	20	10	3.24	Forward	25,382,540	24,637,761	6,875,769	6,874,514	28,384,486
				Reverse	25,382,540	24,566,236	21,529,362	21,509,972	
	30	10	5.80	Forward	17,850,506	15,510,465	8,774,046	8,770,546	22,910,507
				Reverse	17,850,506	15,449,164	14,163,816	14,139,961	
PQ2	0	10	93.88	Forward	19,889,808	19,421,893	8,271,093	8,269,676	24,222,998
				Reverse	19,889,808	19,396,526	15,962,487	15,953,322	
	10	30	0.65	Forward	12,058,714	11,538,283	7,642,078	7,411,005	16,748,402
				Reverse	12,058,714	11,534,662	9,694,669	9,337,397	
	20	30	0.75	Forward	12,156,048	12,054,915	7,964,502	7,495,189	16,787,824
				Reverse	12,156,048	12,011,706	9,936,413	9,292,635	
	30	10	1374.27	Forward	22,282,647	21,484,948	10,542,874	10,541,845	29,050,377
				Reverse	22,282,647	21,450,250	18,514,886	18,508,532	
PQ3	0	10	710.17	Forward	36,488,618	33,010,408	28,261,267	28,260,483	52,259,826
				Reverse	36,488,618	33,039,161	24,010,669	23,999,343	
	10	10	8.78	Forward	55,865,409	46,448,107	40,036,641	40,001,730	78,046,650
				Reverse	55,865,409	46,518,510	38,118,906	38,044,920	
	20	10	1.89	Forward	21,686,715	19,002,297	16,178,519	16,161,512	30,114,310
				Reverse	21,686,715	19,052,553	14,512,067	13,952,798	
	30	10	2.69	Forward	61,688,485	56,117,687	47,841,469	47,835,733	90,382,967
				Reverse	61,688,485	56,043,853	42,934,409	42,547,234	

Table S2. Geochemical and physicochemical properties of the Polloquere soils and spring water.

Transect	Sample	pH	SWC*	Acetate	Cl^-	SO_4^{2-}	NO_3^-	Li^+	Na^+	NH_4^+	K^+	Mg^{2+}	Ca^{2+}
PQ1	0-m	3.13 (0.03)	21.8%	BD	535 (210)	8,355 (372)	BD	3.1 (0.9)	644 (196)	31.3 (9.7)	179 (42)	39 (14)	3,045 (195)
	10-m	4.67 (0.04)	10.9%	BD	7,676 (949)	8,846 (456)	BD	39.9 (3.6)	3,569 (381)	61.6 (1.8)	1,029 (52)	114 (9)	3,230 (362)
	20-m	3.50 (0.02)	17.5%	BD	3,182 (1213)	691 (35)	BD	20.9 (5.9)	1,714 (552)	38.9 (2.1)	628 (221)	54 (11)	346 (56)
	30-m	3.59 (0.02)	18.3%	BD	3,541 (591)	698 (51)	BD	32.0 (20.6)	2,472 (1,329)	42.9 (6.2)	788 (296)	78 (34)	382 (48.4)
PQ2	0-m	5.18 (0.03)	25.8%	28.8 (4.5)	910 (340)	2,178 (592)	54.4 (18.0)	5.52 (1.6)	969 (329)	53.5 (8.2)	385 (125)	50 (15)	865 (246)
	10-m	3.97 (0.01)	14.3%	23.3 (0.8)	94 (7)	2,776 (413)	80.8 (24.0)	1.3 (0.3)	241 (81)	42.0 (19.4)	158 (8)	22 (1)	1,260 (133)
	20-m	4.25 (0.02)	10.5%	24.9 (6.2)	176 (27)	1,821 (75)	9.5 (2.0)	2.2 (0.6)	496 (86)	65.7 (10.9)	165 (30)	51 (4)	788 (35)
	30-m	4.13 (0.02)	7.0%	41.4 (26.2)	27 (13)	201 (76)	37.3 (14.3)	0.5 (0.01)	271 (272)	51.3 (18.5)	63 (2)	16 (4)	269 (49)
PQ3	0-m	2.57 (0.05)	45.1%	24.2 (2.9)	600 (167)	2,701 (428)	12.8 (6.6)	3.2 (0.3)	691 (23)	14.3 (2.0)	262 (20)	76 (10)	588 (82)
	10-m	3.82 (0.01)	18.1%	20.4 (3.5)	5,183 (394)	9,187 (556)	13.5 (6.0)	20.1 (1.9)	3,553 (315)	18.3 (3.0)	830 (72)	115 (10)	3,663 (221)
	20-m	8.66 (0.02)	26.8%	15.9 (1.1)	15,719 (819)	13,867 (140)	16.3 (4.4)	78.5 (5.8)	14,310 (1,133)	18.4 (4.6)	2,153 (147)	611 (32)	4,263 (557)
	30-m	8.43 (0.01)	25.0%	22.4 (3.6)	2,634 (227)	9,642 (722)	25.7 (1.4)	14.4 (1.3)	2,504 (194)	7.7 (0.9)	508 (36)	173 (19)	4,401 (602)
Spring water		8.81 (0.08)	NA	BD	746 (290)	851 (14)	28.1 (11.1)	2.3 (0.4)	670 (137)	28.2 (11.9)	104 (34)	21 (2)	295 (46)

Concentrations reported in mg kg^{-1} soil or mg L^{-1} water; error provided in parentheticals

**Gravimetric soil water content*

BD, below detection limit

Table S3. Soil carbon and nitrogen analyses

Transect	Distance (m)	TIC (%)	$\delta^{13}C_{TIC}$	TOC (%)	$\delta^{13}C_{TOC}$	TN (%)	$\delta^{15}N$	C:N
PQ1	0	0.02	-15.7	0.31	-16.8	0.039	0.3	8.4
PQ1	10	0.04	-23.4	0.18	-22.3	0.016	3.3	13.7
PQ1	20	0.05	-24.2	0.39	-22.3	0.020	2.3	21.5
PQ1	30	0.10	-24.2	0.63	-22.8	0.024	6.1	30.4
PQ2	0	0.02	-21.4	0.15	-18.8	0.022	-0.8	7.8
PQ2	10	BD	BD	0.07	-22.1	0.010	3.0	8.0
PQ2	20	BD	BD	0.13	-23.0	0.014	4.0	8.2
PQ2	30	0.15	-24.2	0.44	-23.4	0.038	4.2	15.4
PQ3	0	0.02	-18.1	0.16	-21.0	0.032	-4.4	5.5
PQ3	10	BD	BD	0.54	-20.0	0.056	-1.4	9.6
PQ3	20	0.15	-7.3	1.21	-19.7	0.100	6.6	13.6
PQ3	30	0.10	-20.6	0.92	-21.8	0.079	1.9	12.9

TIC, total inorganic carbon; TOC, total organic carbon; TN, total nitrogen
BD, below detection

Table S4. MAGs data and statistics

Bin	Classification							Complete-ness[a] (%)	Contamin-ation[a] (%)	Contig count	Assembly size (bp)	Mapped reads	GC content (%)	N50	Relative Abund-ance[b] (%)
	Domain	Phylum	Class	Order	Family	Genus	Species								
PQ1-0S_bin_1	Bacteria	SZUA-182	SZUA-182	DTGQ01	DTGQ01	DTGQ01		79.0	3.4	648	4,586,725	349,748	49.4	8,135	5.5
PQ1-10S_bin_2	Bacteria	Actinobacteriota	Acidimicrobiia	Acidimicrobiales	QHCF01			99.7	1.3	72	2,601,244	899,611	70.9	47,115	22.4
PQ1-10S_bin_1	Bacteria	Actinobacteriota	Thermoleophilia	Gaiellales	Gaiellaceae	Palsa-739		92.9	0.9	414	2,771,148	239,516	72.0	10,074	6.0
PQ1-10S_bin_3	Bacteria	Actinobacteriota	Acidimicrobiia	Acidimicrobiales	RAAP-2	PALSA-457		90.6	4.3	465	2,898,152	267,374	73.7	8,033	6.7
PQ1-20S_bin_2	Bacteria	Actinobacteriota	Actinomycetia	Mycobacteriales	Mycobacteriaceae	Mycobacterium		99.4	0.6	212	4,308,548	1,854,284	65.9	40,648	6.5
PQ1-20S_bin_1	Bacteria	Actinobacteriota	Actinomycetia	Mycobacteriales	Mycobacteriaceae	Mycobacterium		96.7	0.5	196	3,446,448	14,882,404	69.0	16,649	52.4
PQ1-20S_bin_3	Bacteria	Actinobacteriota	Actinomycetia	Mycobacteriales	Mycobacteriaceae	Mycobacterium		89.4	1.2	627	4,432,521	329,415	67.4	10,025	1.2
PQ2-0S_bin_18	Bacteria	Proteobacteria	Zetaproteobacteria	Mariprofundales	Mariprofundaceae	Mariprofundus		100.0	0.0	195	2,606,007	768,687	54.1	19,096	3.2
PQ2-0S_bin_2	Bacteria	Proteobacteria	Gammaproteobacteria	Burkholderiales	Gallionellaceae	Gallionella		98.9	1.4	110	2,993,481	696,151	55.7	42,166	2.9
PQ2-0S_bin_24	Bacteria	Campylobacterota	Campylobacteria	Campylobacterales	Sulfurimonadaceae	Sulfurimonas		98.8	2.5	214	2,310,629	157,177	34.5	15,862	0.6
PQ2-0S_bin_8	Bacteria	Desulfobacterota	Desulfuromonadia	Desulfuromonadales	BM103			98.7	0.0	182	3,727,201	326,071	56.0	34,909	1.3
PQ2-0S_bin_17	Bacteria	Firmicutes	Bacilli	Acholeplasmatales	Acholeplasmataceae	UBA2284		98.7	2.2	42	1,670,486	149,652	39.3	89,663	0.6
PQ2-0S_bin_7	Bacteria	Campylobacterota	Campylobacteria	Campylobacterales	Sulfurimonadaceae	CAITKP01		97.6	3.5	132	2,173,976	256,047	37.7	22,754	1.1
PQ2-0S_bin_1	Bacteria	Proteobacteria	Gammaproteobacteria	SZUA-229	SZUA-229			97.5	1.7	119	3,022,166	1,128,724	49.4	45,153	4.7
PQ2-0S_bin_19	Bacteria	Bacteroidota	Bacteroidia	Bacteroidales	Prolixibacteraceae	Draconibacterium		95.7	4.1	359	4,540,232	602,441	40.7	18,993	2.5
PQ2-0S_bin_12	Bacteria	Planctomycetota	Phycisphaerae	Sedimentisphaerales	Anaerohalosphaeraceae	4572-13		92.6	2.3	526	4,607,691	263,269	51.6	11,704	1.1
PQ2-0S_bin_22	Bacteria	Proteobacteria	Gammaproteobacteria	SZUA-229	SZUA-229			89.9	0.5	218	2,507,272	297,918	49.1	15,420	1.2
PQ2-0S_bin_13	Bacteria	Proteobacteria	Alphaproteobacteria	Parvibaculales	Parvibaculaceae	Parvibaculum		89.6	2.1	734	3,249,826	134,997	58.3	5,512	0.6
PQ2-0S_bin_11	Bacteria	Proteobacteria	Gammaproteobacteria	SLND01	SLND01			83.3	1.9	229	2,391,151	325,792	61.9	17,514	1.3
PQ2-0S_bin_14	Bacteria	Acidobacteriota	Acidobacteriae	Acidobacteriales	Acidobacteriaceae	Acidobacterium		83.0	1.9	650	3,072,040	130,682	61.8	5,831	0.5
PQ2-0S_bin_6	Bacteria	Bacteroidota	Bacteroidia	Bacteroidales	Prolixibacteraceae	Prolixibacter		82.7	4.2	707	3,366,539	158,277	44.6	5,348	0.7
PQ2-0S_bin_16	Bacteria	Patescibacteria	Paceibacteria	UBA9983_A	UBA9973	UBA8515		79.9	1.1	39	690,937	80,616	31.4	32,946	0.3
PQ2-0S_bin_15	Bacteria	Actinobacteriota	Actinomycetia	Mycobacteriales	Mycobacteriaceae	Mycobacterium	Mycobacterium helveticum	79.8	4.2	1037	4,259,307	238,186	69.0	4,826	1.0
PQ2-0S_bin_4	Bacteria	Bacteroidota	Bacteroidia	Bacteroidales	UBA12170	UBA12170		78.5	0.4	521	2,160,187	128,423	35.7	4,792	0.5
PQ2-0S_bin_21	Bacteria	Bacteroidota	Bacteroidia	Bacteroidales	F082	SZUA-53		77.3	2.2	481	3,363,094	341,679	39.5	7,918	1.4
PQ2-0S_bin_25	Bacteria	Elusimicrobiota	Elusimicrobia	Elusimicrobiales	UBA9959	UBA9959		72.7	2.3	706	2,460,205	100,799	57.0	4,034	0.4
PQ2-0S_bin_23	Bacteria	Proteobacteria	Gammaproteobacteria	Halothiobacillales	Halothiobacillaceae	Halothiobacillus		68.3	1.7	505	1,725,275	63,637	55.0	3,858	0.3
PQ2-0S_bin_10	Bacteria	Bacteroidota	Bacteroidia	Bacteroidales				63.9	0.8	1069	3,366,912	117,499	40.7	3,326	0.5
PQ2-0S_bin_20	Bacteria	Patescibacteria	JAEDAM01	Absconditabacterales	X112	UBA5124		63.8	0.0	254	1,026,930	45,782	28.6	4,791	0.2
PQ2-0S_bin_5	Bacteria	Bipolaricaulota	Bipolaricaulia	UBA7950	UBA9294			63.1	1.7	576	1,676,230	57,011	64.3	2,981	0.2
PQ2-0S_bin_9	Bacteria	Patescibacteria	ABY1	BM507	UBA12465	JAACPL01		60.2	1.5	158	679,393	36,856	33.3	5,151	0.2
PQ2-10S_bin_1	Bacteria	Proteobacteria	Alphaproteobacteria	Acetobacterales	Acetobacteraceae	Acidiphilium		86.7	2.0	663	2,348,297	170,749	66.7	3,940	1.0
PQ2-10S_bin_7	Bacteria	Actinobacteriota	Acidimicrobiia	Acidimicrobiales	RAAP-2	RAAP-2		86.5	0.5	366	1,740,882	89,125	59.4	5,835	0.5
PQ2-10S_bin_3	Bacteria	Chloroflexota	UBA4733	UBA4733	UBA4733	UBA4733		75.0	3.0	1091	3,423,829	285,014	66.3	3,303	1.7
PQ2-10S_bin_4	Bacteria	Actinobacteriota	Acidimicrobiia	Acidimicrobiales				70.0	0.9	761	2,973,949	377,677	67.4	3,699	2.3
PQ2-10S_bin_2	Bacteria	Actinobacteriota	Acidimicrobiia	Acidimicrobiales	Bog-793			63.1	1.6	501	1,503,592	61,793	54.1	3,169	0.4
PQ2-10S_bin_6	Bacteria	Chloroflexota	UBA5177	UBA5177	UBA5177			57.5	5.4	970	3,065,079	203,265	60.5	3,331	1.2
PQ2-20S_bin_5	Bacteria	Actinobacteriota	Acidimicrobiia	Acidimicrobiales	QHCF01			97.4	2.1	203	3,235,442	484,977	67.8	24,976	2.9
PQ2-20S_bin_7	Bacteria	Firmicutes_E	Sulfobacillia	Sulfobacillales	Sulfobacillaceae			95.7	1.3	460	2,991,820	197,431	56.4	10,185	1.2
PQ2-20S_bin_1	Bacteria	Actinobacteriota	Acidimicrobiia	Acidimicrobiales				93.6	0.9	529	2,827,232	228,756	73.0	6,782	1.4
PQ2-20S_bin_3	Bacteria	Chloroflexota	UBA5177	UBA5177	UBA5177			91.7	1.0	300	2,805,714	1,344,902	63.1	13,453	8.0
PQ2-20S_bin_6	Bacteria	Actinobacteriota	Actinomycetia	Mycobacteriales	Pseudonocardiaceae			89.2	1.4	237	3,201,855	284,571	71.8	18,840	1.7
PQ2-20S_bin_4	Bacteria	Proteobacteria	Gammaproteobacteria	Steroidobacterales	Steroidobacteraceae	13-2-20CM-66-19		60.1	2.0	712	2,270,637	94,609	67.9	3,405	0.6
PQ2-20S_bin_8	Bacteria	Firmicutes	Bacilli	Alicyclobacillales	Alicyclobacillaceae			55.5	0.0	405	1,334,864	55,825	57.7	3,444	0.3
PQ2-20S_bin_2	Bacteria	Actinobacteriota	Actinomycetia	Streptosporangiales	Streptosporangiaceae	Trebonia		51.5	3.3	1017	4,627,498	245,828	69.4	4,974	1.5
PQ2-20S_bin_9	Bacteria	Chloroflexota	UBA4733	UBA4733	UBA4733			50.5	9.6	2117	4,338,369	172,207	63.9	1,974	1.0
PQ2-30S_bin_2	Bacteria	Actinobacteriota	Acidimicrobiia	Acidimicrobiales	QHCF01	QHCF01		97.0	1.3	67	2,565,230	762,328	70.8	54,758	2.6
PQ2-30S_bin_17	Bacteria	Actinobacteriota	Thermoleophilia	Solirubrobacterales	Solirubrobacteraceae	Palsa-465		96.7	0.1	176	3,014,664	345,742	68.5	24,152	1.2
PQ2-30S_bin_7	Bacteria	Chloroflexota	UBA5177	UBA5177	UBA5177			95.4	1.7	309	4,753,862	305,907	65.5	20,639	1.1
PQ2-30S_bin_8	Bacteria	Actinobacteriota	Thermoleophilia	Gaiellales	Gaiellaceae	Palsa-739		92.2	0.9	230	2,727,795	311,551	71.2	16,638	1.1
PQ2-30S_bin_12	Bacteria	Chloroflexota	UBA5177	UBA5177	UBA5177			89.1	3.2	458	2,906,299	216,866	63.0	7,913	0.7
PQ2-30S_bin_6	Bacteria	Actinobacteriota	Actinomycetia	Streptosporangiales	Streptosporangiaceae	Bog-532		77.0	7.0	2087	7,886,639	550,592	70.6	4,163	1.9
PQ2-30S_bin_3	Bacteria	Proteobacteria	Alphaproteobacteria	ATCC43930	Stellaceae	AP-15		69.0	2.8	1362	4,390,561	189,327	65.9	3,459	0.7
PQ2-30S_bin_4	Bacteria	Actinobacteriota	Actinomycetia	Mycobacteriales	Pseudonocardiaceae			65.5	2.6	419	1,829,850	86,706	72.7	5,592	0.3
PQ2-30S_bin_9	Bacteria	Actinobacteriota	Thermoleophilia	Solirubrobacterales	Solirubrobacteraceae	Palsa-465		62.7	2.0	351	2,059,527	113,484	71.0	6,517	0.4
PQ2-30S_bin_14	Bacteria	Actinobacteriota	Thermoleophilia	Solirubrobacterales	Solirubrobacteraceae	Palsa-465		62.0	0.0	231	2,622,809	214,539	68.7	13,687	0.7
PQ2-30S_bin_5	Bacteria	Acidobacteriota	Acidobacteriae	Bryobacterales	Bryobacteraceae	Bog-105		61.9	0.0	685	3,395,037	232,071	58.1	5,867	0.8
PQ2-30S_bin_11	Bacteria	Chloroflexota	Ktedonobacteria	Ktedonobacterales	Ktedonobacteraceae	CADDZH01		60.8	6.1	826	3,761,020	141,983	57.0	4,888	0.5
PQ2-30S_bin_16	Bacteria	Chloroflexota	UBA5177	UBA5177				59.7	1.0	354	1,837,342	74,595	61.7	5,440	0.3
PQ2-30S_bin_10	Bacteria	Planctomycetota	Planctomycetia	Gemmatales	Gemmataceae			59.7	2.3	1390	5,056,589	211,100	61.5	4,102	0.7
PQ2-30S_bin_1	Bacteria	Actinobacteriota	Actinomycetia	Mycobacteriales	Mycobacteriaceae	Mycobacterium		53.7	2.3	1009	3,453,896	150,977	64.9	3,935	0.5
PQ3-0S_bin_5	Bacteria	Campylobacterota	Campylobacteria	Campylobacterales	Sulfurimonadaceae	Sulfuricurvum		100.0	0.8	20	2,566,042	235,099	44.2	204,719	0.4
PQ3-0S_bin_9	Bacteria	Bacteroidota	Bacteroidia	Bacteroidales	Paludibacteraceae	Microbacter		98.8	0.0	181	2,707,092	210,972	41.0	25,077	0.4
PQ3-0S_bin_24	Bacteria	Bacteroidota	Bacteroidia	Bacteroidales	Williamwhitmaniaceae	Williamwhitmania		98.8	4.0	209	3,767,587	1,252,970	44.4	28,858	2.4
PQ3-0S_bin_8	Bacteria	Firmicutes	Bacilli	Acholeplasmatales	Acholeplasmataceae	UBA2284		98.7	0.9	24	1,703,785	173,913	39.2	114,412	0.3
PQ3-0S_bin_7	Bacteria	Bacteroidota	Ignavibacteria	Ignavibacteriales	Ignavibacteriaceae	Ignavibacterium		98.5	0.6	152	2,841,163	822,076	33.4	22,387	1.6
PQ3-0S_bin_10	Bacteria	Bacteroidota	Bacteroidia	Bacteroidales	S143-33	JAFGUD01		98.4	1.1	152	3,524,963	400,729	28.9	31,980	0.8
PQ3-0S_bin_13	Bacteria	Proteobacteria	Gammaproteobacteria	Burkholderiales	Thiobacillaceae	Thiobacillus		97.9	1.3	162	2,326,354	814,080	65.7	24,065	1.6
PQ3-0S_bin_15	Bacteria	Proteobacteria	Gammaproteobacteria	Halothiobacillales	Halothiobacillaceae	Halothiobacillus		97.8	0.0	167	2,726,046	452,365	54.7	29,689	0.9
PQ3-0S_bin_16	Bacteria	Desulfobacterota	Desulfuromonadia	Desulfuromonadales	Desulfuromonadaceae	Pseudodesulfuromonas	Pseudodesulfuromonas thiophila	96.8	1.3	100	2,283,485	189,037	61.6	34,186	0.4
PQ3-0S_bin_1	Bacteria	Campylobacterota	Campylobacteria	Campylobacterales	Sulfurospirillaceae	JAADIZ01		96.4	1.7	168	2,037,782	145,860	36.6	17,657	0.3
PQ3-0S_bin_25	Bacteria	Proteobacteria	Gammaproteobacteria	SpSt-1174	SpSt-1174			94.8	1.2	76	2,788,341	520,385	68.5	46,781	1.0
PQ3-0S_bin_17	Bacteria	Caldisericota	Caldisericia	Cryosericales	Cryosericaceae	Cryosericum		94.6	0.0	36	1,585,083	492,205	56.5	71,574	0.9
PQ3-0S_bin_12	Bacteria	Proteobacteria	Gammaproteobacteria	Burkholderiales	Thiobacillaceae	UBA3361		94.5	1.5	328	2,396,223	5,255,208	65.5	9,511	10.1
PQ3-0S_bin_2	Bacteria	Bacteroidota	Ignavibacteria	Ignavibacteriales	Ignavibacteriaceae			93.0	0.0	183	2,784,180	3,553,346	33.0	23,198	6.8
PQ3-0S_bin_3	Bacteria	Spirochaetota	Spirochaetia	Treponematales	Termitinemataceae			92.5	2.3	608	2,842,442	140,436	52.9	5,662	0.3
PQ3-0S_bin_19	Bacteria	Chloroflexota	Anaerolineae	CG2-30-64-16	CG2-30-64-16	MWBF01		89.1	3.4	265	4,170,585	878,752	64.4	19,120	1.7
PQ3-0S_bin_11	Bacteria	Bacteroidota	Bacteroidia	Bacteroidales	Prolixibacteraceae	UBA6024		89.0	4.6	891	4,919,546	206,757	43.2	6,731	0.4
PQ3-0S_bin_6	Bacteria	Deferribacterota	Deferribacteres	Deferribacterales	Calditerrivibrionaceae			88.7	2.8	504	1,744,440	128,244	36.1	3,765	0.2
PQ3-0S_bin_14	Bacteria	Proteobacteria	Gammaproteobacteria	Burkholderiales	Thiobacillaceae	Thiobacillus		84.1	2.2	366	1,915,859	152,335	65.1	6,736	0.3
PQ3-0S_bin_27	Bacteria	Chloroflexota	Anaerolineae	Anaerolineales	Anaerolineaceae	UBA6107		82.1	6.0	495	1,931,096	124,340	52.5	4,593	0.2
PQ3-0S_bin_18	Bacteria	Acidobacteriota	Acidobacteriae	Bryobacterales	Bryobacteraceae			76.3	2.3	1032	3,026,013	136,828	66.4	3,184	0.3
PQ3-0S_bin_22	Bacteria	Bacteroidota	Bacteroidia	Bacteroidales	GWA2-32-17	Ch61		67.1	2.8	539	1,808,949	72,349	34.9	3,697	0.1
PQ3-0S_bin_26	Bacteria	Patescibacteria	Saccharimonadia	Saccharimonadales	UBA1547	UBA1547		59.6	0.9	124	698,379	29,973	41.7	7,629	0.1
PQ3-0S_bin_21	Bacteria	Desulfobacterota	Desulfobacteria	Desulfobacterales	Desulfatirhabdiaceae	Desulfatirhabdium		58.8	0.9	677	2,294,895	124,918	54.5	3,692	0.2
PQ3-0S_bin_20	Archaea	Nanoarchaeota	Nanoarchaeia	Woesearchaeales	UBA9642	UBA9642		58.3	0.9	247	839,303	31,601	33.5	3,681	0.1
PQ3-0S_bin_4	Bacteria	Chloroflexota	Anaerolineae	Anaerolineales	Anaerolineaceae	Bellilinea		52.0	1.8	381	1,164,879	52,043	50.8	3,158	0.1
PQ3-0S_bin_28	Bacteria	Spirochaetota	Spirochaetia	Treponematales	UBA12059			51.6	2.5	736	1,753,070	63,920	58.8	2,311	0.1
PQ3-10S_bin_2	Bacteria	Proteobacteria	Gammaproteobacteria	Acidithiobacillales	Acidithiobacillaceae	Acidithiobacillus	Acidithiobacillus sp018853445	99.3	1.9	94	2,911,073	6,986,631	52.1	60,687	9.0
PQ3-10S_bin_7	Bacteria	Proteobacteria	Alphaproteobacteria	Acetobacterales	Acetobacteraceae	Acidiphilium		98.3	0.2	212	3,283,607	678,874	65.4	30,315	0.9
PQ3-10S_bin_5	Bacteria	Actinobacteriota	Actinomycetia	Mycobacteriales	Mycobacteriaceae	Mycobacterium		98.1	0.0	203	3,527,730	1,691,280	69.1	18,651	2.2
PQ3-10S_bin_9	Bacteria	Acidobacteriota	Acidobacteriae	Acidobacteriales	Acidobacteriaceae	Acidobacterium		97.4	1.7	336	4,558,294	1,060,954	60.4	24,620	1.4
PQ3-10S_bin_6	Bacteria	Actinobacteriota	Actinomycetia	Mycobacteriales	Mycobacteriaceae	Mycobacterium		95.6	6.3	219	4,032,064	356,388	66.3	24,467	0.5
PQ3-10S_bin_11	Bacteria	Actinobacteriota	Acidimicrobiia	Acidimicrobiales	Palsa-688			95.1	1.3	176	3,246,527	1,282,795	71.7	15,610	1.6
PQ3-10S_bin_1	Bacteria	Firmicutes	Bacilli	Alicyclobacillales	Alicyclobacillaceae	Alicyclobacillus		93.8	0.7	533	3,444,459	241,001	48.9	8,694	0.3
PQ3-10S_bin_8	Bacteria	Actinobacteriota	Actinomycetia	Mycobacteriales	Mycobacteriaceae	Mycobacterium		90.7	3.0	192	3,963,689	277,112	68.7	28,030	0.4
PQ3-10S_bin_13	Bacteria	Proteobacteria	Gammaproteobacteria	REEB76	REEB76			90.0	0.6	134	2,079,735	9,326,615	62.0	21,104	12.0
PQ3-10S_bin_15	Bacteria	Proteobacteria	Alphaproteobacteria	Acetobacterales	Acetobacteraceae			88.3	4.4	634	3,037,138	216,039	67.1	6,301	0.3
PQ3-10S_bin_12	Bacteria	Proteobacteria	Gammaproteobacteria	Nevskiales	Nevskiaceae	SCRT01		77.8	2.3	493	2,052,868	106,142	65.4	4,799	0.1
PQ3-10S_bin_3	Bacteria	Proteobacteria	Gammaproteobacteria	Steroidobacterales	Steroidobacteraceae			72.6	0.0	492	2,317,194	145,258	67.1	5,641	0.2
PQ3-10S_bin_4	Bacteria	Proteobacteria	Alphaproteobacteria	Acetobacterales	Acetobacteraceae	Acidiphilium		70.1	6.5	1067	3,384,656	154,368	62.9	3,488	0.2
PQ3-10S_bin_14	Bacteria	Proteobacteria	Alphaproteobacteria	Rickettsiales	Midichloriaceae	Jidaibacter		64.4	0.1	419	1,113,414	43,546	32.6	2,723	0.1
PQ3-20S_bin_2	Bacteria	Proteobacteria	Gammaproteobacteria	Arenicellales	JAANXG01	JAANXG01		98.2	1.7	150	3,855,953	699,574	63.8	40,642	2.3
PQ3-20S_bin_1	Bacteria	Proteobacteria	Gammaproteobacteria	HK1	HK1			58.9	3.5	826	2,448,688	173,600	63.5	3,165	0.6
PQ3-20S_bin_3	Bacteria	Proteobacteria	Gammaproteobacteria	SLND01	SLND01			52.4	2.1	390	1,028,372	64,517	65.7	2,684	0.2
PQ3-30S_bin_6	Bacteria	Proteobacteria	Gammaproteobacteria	Pseudomonadales	Halieaceae	Kineobactrum		100.0	1.5	49	5,456,969	794,526	61.2	169,285	0.9
PQ3-30S_bin_5	Bacteria	Bacteroidota	Bacteroidia	Flavobacteriales	Flavobacteriaceae	Sinomicrobium		99.0	1.6	89	5,374,563	1,713,841	43.6	175,643	1.9
PQ3-30S_bin_4	Bacteria	Proteobacteria	Gammaproteobacteria	Pseudomonadales	Cellvibrionaceae	Cellvibrio		98.3	0.1	132	5,173,874	1,227,322	49.5	50,708	1.4
PQ3-30S_bin_2	Bacteria	KSB1	UBA2214	DRLW01	DRLW01			96.6	1.3	508	6,002,978	417,011	52.8	16,922	0.5
PQ3-30S_bin_10	Bacteria	Actinobacteriota	UBA4738	UBA4738				94.3	0.9	373	1,807,952	114,475	71.0	5,812	0.1
PQ3-30S_bin_12	Bacteria	Proteobacteria	Gammaproteobacteria	Woeseiales	Woeseiaceae	UBA1844		86.9	2.4	250	3,057,528	267,976	65.6	21,379	0.3
PQ3-30S_bin_8	Bacteria	Proteobacteria	Alphaproteobacteria	Kiloniellales	Kiloniellaceae	WHTV01		60.5	4.3	892	3,248,946	163,203	67.8	4,070	0.2
PQ3-30S_bin_9	Bacteria	Krumholzibacteriota	Krumholzibacteria					57.5	0.0	778	2,423,656	101,369	67.7	3,317	0.1
PQ3-30S_bin_13	Bacteria	Proteobacteria	Gammaproteobacteria	Woeseiales	Woeseiaceae	SZUA-117		56.1	2.2	972	2,159,585	92,588	60.9	2,198	0.1
PQ3-30S_bin_11	Bacteria	Proteobacteria	Gammaproteobacteria	Burkholderiales	SG8-39	JAABQT01		55.8	4.4	635	1,681,575	91,468	70.1	2,726	0.1
PQ3-30S_bin_3	Bacteria	Proteobacteria	Gammaproteobacteria	Competibacterales	Competibacteraceae			53.8	3.4	875	2,545,338	243,019	65.6	3,038	0.3
PQ3-30S_bin_7	Bacteria	Acidobacteriota	Thermoanaerobaculia	UBA5704				51.3	1.7	960	2,547,874	130,227	72.5	2,748	0.1

[a]Determined by CheckM

[b]Percentage of reads mapped to the bin in the metagenome

Table S5. MAG metabolic pathways

Bin	[NiFe]-Hydrogenases[b]	CoxL?[c]	SULFUR: Thiosulfate Oxidation	SULFUR: Sulfide Oxidation	SULFUR: Sulfate Reduction/ Sulfur Oxidation	NITROGEN: DNR	NITROGEN: NO_2^- reduction	NITROGEN: NO reduction	NITROGEN: N_2O reduction	NITROGEN: Ammonification	NITROGEN: NH_3 oxidation	OXYGEN: Aerobic respiration	OXYGEN: Microaerobic respiration	CARBON: rTCA	CARBON: WL	CARBON: CBB
			CONFIRMED METABOLISMS[a]													
PQ1-0S_bin_1	1b, 3b, 3b, 4g	FALSE	FALSE	FALSE	FALSE	FALSE	FALSE	FALSE	FALSE	FALSE	FALSE	FALSE	FALSE	FALSE	FALSE	FALSE
PQ1-10S_bin_2	4b, 4f, 4g	FALSE	FALSE	FALSE	FALSE	FALSE	FALSE	FALSE	FALSE	FALSE	FALSE	TRUE	FALSE	FALSE	FALSE	FALSE
PQ1-10S_bin_3	4f, 4g	TRUE	FALSE	FALSE	FALSE	FALSE	FALSE	FALSE	FALSE	FALSE	FALSE	TRUE	FALSE	FALSE	FALSE	FALSE
PQ1-10S_bin_1	4b, 4f, 4f, 4f, 4g	FALSE	FALSE	FALSE	FALSE	FALSE	FALSE	FALSE	FALSE	FALSE	FALSE	TRUE	FALSE	FALSE	FALSE	FALSE
PQ1-20S_bin_2	1h, 3b, 4g	FALSE	FALSE	FALSE	FALSE	FALSE	FALSE	FALSE	FALSE	FALSE	FALSE	TRUE	TRUE	FALSE	FALSE	FALSE
PQ1-20S_bin_1	1h, 4f, 4g	TRUE	FALSE	FALSE	FALSE	TRUE	FALSE	FALSE	FALSE	TRUE	FALSE	FALSE	TRUE	FALSE	FALSE	FALSE
PQ1-20S_bin_3	3b, 4b, 4e, 4f, 4g	FALSE	FALSE	FALSE	FALSE	FALSE	FALSE	FALSE	FALSE	FALSE	FALSE	TRUE	TRUE	FALSE	FALSE	TRUE
PQ2-0S_bin_2	1e, 3d, 4f, 4g	FALSE	TRUE	FALSE	TRUE	FALSE	FALSE	FALSE	FALSE	TRUE	FALSE	FALSE	TRUE	FALSE	FALSE	TRUE
PQ2-0S_bin_1	1e, 3d, 4e	FALSE	TRUE	FALSE	TRUE	FALSE	FALSE	FALSE	TRUE	TRUE	FALSE	TRUE	TRUE	FALSE	FALSE	TRUE
PQ2-0S_bin_8	1f, 3b, 4b, 4b, 4f, 4f, 4g	FALSE	FALSE	FALSE	FALSE	FALSE	FALSE	FALSE	FALSE	FALSE	FALSE	TRUE	TRUE	FALSE	FALSE	FALSE
PQ2-0S_bin_17	4g, 4g	FALSE	FALSE	FALSE	FALSE	FALSE	FALSE	FALSE	FALSE	FALSE	FALSE	FALSE	TRUE	FALSE	FALSE	FALSE
PQ2-0S_bin_11	4f, 4g	TRUE	TRUE	FALSE	FALSE	FALSE	FALSE	FALSE	FALSE	FALSE	FALSE	TRUE	TRUE	FALSE	FALSE	FALSE
PQ2-0S_bin_19	4b, 4e, 4f	TRUE	FALSE	FALSE	FALSE	TRUE	FALSE	FALSE	FALSE	FALSE	FALSE	TRUE	TRUE	FALSE	FALSE	FALSE
PQ2-0S_bin_7	1b, 2d, 4e, 4e	FALSE	TRUE	FALSE	FALSE	FALSE	FALSE	FALSE	FALSE	FALSE	FALSE	FALSE	TRUE	TRUE	FALSE	FALSE
PQ2-0S_bin_22	1e, 3d, 4e	FALSE	TRUE	FALSE	TRUE	FALSE	TRUE	FALSE	TRUE	FALSE	FALSE	TRUE	TRUE	FALSE	FALSE	TRUE
PQ2-0S_bin_18	3d, 4b, 4g	FALSE	FALSE	FALSE	FALSE	FALSE	TRUE	FALSE	FALSE	FALSE	FALSE	FALSE	TRUE	FALSE	FALSE	TRUE
PQ2-0S_bin_24	1b, 1b, 2d, 2d, 4e, 4g, 4g	FALSE	TRUE	FALSE	FALSE	FALSE	FALSE	FALSE	FALSE	FALSE	FALSE	FALSE	TRUE	TRUE	FALSE	FALSE
PQ2-0S_bin_21	1d, 3d, 4b, 4e, 4f, 4g, 4g, 4g	FALSE	FALSE	FALSE	FALSE	TRUE	FALSE	FALSE	FALSE	FALSE	FALSE	TRUE	TRUE	FALSE	FALSE	FALSE
PQ2-0S_bin_12	1a, 1a, 3b, 4b, 4f	FALSE	FALSE	FALSE	FALSE	FALSE	FALSE	FALSE	FALSE	FALSE	FALSE	FALSE	TRUE	FALSE	FALSE	FALSE
PQ2-0S_bin_15	1h, 3d, 4f, 4f, 4g	TRUE	FALSE	FALSE	FALSE	TRUE	FALSE	FALSE	FALSE	TRUE	FALSE	FALSE	TRUE	FALSE	FALSE	FALSE
PQ2-0S_bin_6	4e, 4e, 4f	TRUE	FALSE	FALSE	FALSE	TRUE	FALSE	FALSE	FALSE	FALSE	FALSE	TRUE	TRUE	FALSE	FALSE	FALSE
PQ2-0S_bin_13	4g	FALSE	FALSE	FALSE	FALSE	FALSE	FALSE	FALSE	FALSE	FALSE	FALSE	TRUE	TRUE	FALSE	FALSE	FALSE
PQ2-0S_bin_23	4e	FALSE	TRUE	TRUE	FALSE	FALSE	FALSE	FALSE	FALSE	FALSE	FALSE	FALSE	TRUE	FALSE	FALSE	FALSE
PQ2-0S_bin_14	1f, 4b, 4g, 4g	FALSE	TRUE	FALSE	FALSE	FALSE	FALSE	FALSE	FALSE	FALSE	FALSE	FALSE	FALSE	FALSE	FALSE	FALSE
PQ2-0S_bin_4	4e, 4f, 4f	FALSE	FALSE	FALSE	FALSE	FALSE	FALSE	FALSE	FALSE	FALSE	FALSE	FALSE	TRUE	FALSE	FALSE	FALSE
PQ2-0S_bin_20	NULL	FALSE	FALSE	FALSE	FALSE	FALSE	FALSE	FALSE	FALSE	FALSE	FALSE	FALSE	FALSE	FALSE	FALSE	FALSE
PQ2-0S_bin_25	3b, 3b, 4a, 4b, 4e, 4f, 4g, 4g	TRUE	FALSE	FALSE	FALSE	FALSE	FALSE	FALSE	FALSE	TRUE	FALSE	FALSE	TRUE	FALSE	FALSE	FALSE
PQ2-0S_bin_10	3d, 4g, 4g, 4g	FALSE	FALSE	FALSE	FALSE	FALSE	FALSE	FALSE	FALSE	FALSE	FALSE	TRUE	TRUE	FALSE	FALSE	FALSE
PQ2-0S_bin_5	4a, 4d	TRUE	FALSE	FALSE	FALSE	FALSE	FALSE	FALSE	FALSE	FALSE	FALSE	FALSE	FALSE	FALSE	FALSE	FALSE
PQ2-10S_bin_7	4g	FALSE	FALSE	FALSE	FALSE	FALSE	FALSE	FALSE	FALSE	FALSE	FALSE	TRUE	FALSE	FALSE	FALSE	FALSE
PQ2-10S_bin_4	1h, 1l, 4b, 4e, 4f	TRUE	FALSE	FALSE	FALSE	FALSE	FALSE	FALSE	FALSE	TRUE	FALSE	TRUE	FALSE	FALSE	FALSE	TRUE
PQ2-10S_bin_1	4b, 4e, 4f	FALSE	TRUE	FALSE	FALSE	FALSE	FALSE	FALSE	FALSE	TRUE	FALSE	TRUE	TRUE	FALSE	FALSE	FALSE
PQ2-10S_bin_2	3b, 4b, 4g	FALSE	FALSE	FALSE	FALSE	FALSE	FALSE	FALSE	FALSE	FALSE	FALSE	FALSE	FALSE	FALSE	FALSE	FALSE
PQ2-10S_bin_6	1h, 1h, 1l, 4b, 4f, 4g, 4g	FALSE	FALSE	FALSE	FALSE	FALSE	FALSE	FALSE	FALSE	FALSE	FALSE	FALSE	FALSE	FALSE	FALSE	FALSE
PQ2-10S_bin_3	4b, 4e	TRUE	FALSE	FALSE	FALSE	FALSE	TRUE	FALSE	FALSE	FALSE	FALSE	FALSE	TRUE	FALSE	FALSE	FALSE
PQ2-20S_bin_5	1h, 1l, 4b, 4f, 4g	FALSE	FALSE	FALSE	FALSE	FALSE	FALSE	FALSE	FALSE	TRUE	FALSE	TRUE	FALSE	FALSE	FALSE	TRUE
PQ2-20S_bin_6	1h, 1h, 1h, 4b, 4e, 4f, 4g	TRUE	FALSE	FALSE	FALSE	FALSE	FALSE	FALSE	FALSE	TRUE	FALSE	FALSE	FALSE	FALSE	FALSE	TRUE
PQ2-20S_bin_1	1l, 4b, 4f, 4g	TRUE	FALSE	FALSE	FALSE	FALSE	FALSE	FALSE	FALSE	TRUE	FALSE	TRUE	FALSE	FALSE	FALSE	TRUE
PQ2-20S_bin_3	4f, 4g	TRUE	FALSE	FALSE	FALSE	FALSE	FALSE	FALSE	FALSE	FALSE	FALSE	FALSE	FALSE	FALSE	FALSE	FALSE
PQ2-20S_bin_4	4b, 4e	FALSE	FALSE	FALSE	FALSE	FALSE	FALSE	FALSE	FALSE	FALSE	FALSE	TRUE	FALSE	FALSE	FALSE	FALSE
PQ2-20S_bin_2	1h	TRUE	FALSE	FALSE	FALSE	FALSE	FALSE	FALSE	FALSE	FALSE	FALSE	FALSE	FALSE	FALSE	FALSE	FALSE
PQ2-20S_bin_7	4b, 4e	FALSE	FALSE	FALSE	FALSE	FALSE	FALSE	FALSE	FALSE	FALSE	FALSE	FALSE	FALSE	FALSE	FALSE	FALSE
PQ2-20S_bin_9	1h, 1h, 1h, 4c, 4f, 4g, 4g	TRUE	FALSE	FALSE	FALSE	FALSE	FALSE	FALSE	FALSE	FALSE	FALSE	FALSE	TRUE	FALSE	FALSE	FALSE
PQ2-30S_bin_2	1h, 4f, 4f, 4g	FALSE	FALSE	FALSE	FALSE	FALSE	FALSE	FALSE	FALSE	TRUE	FALSE	TRUE	FALSE	FALSE	FALSE	TRUE
PQ2-30S_bin_17	1h, 4b, 4e, 4f	TRUE	FALSE	FALSE	FALSE	FALSE	FALSE	FALSE	FALSE	FALSE	FALSE	FALSE	FALSE	FALSE	FALSE	FALSE
PQ2-30S_bin_8	1h, 1h, 4f, 4f, 4g	FALSE	FALSE	FALSE	FALSE	FALSE	FALSE	FALSE	FALSE	FALSE	FALSE	TRUE	FALSE	FALSE	FALSE	FALSE
PQ2-30S_bin_7	4a, 4f, 4f, 4g	TRUE	FALSE	FALSE	FALSE	FALSE	FALSE	FALSE	FALSE	FALSE	FALSE	FALSE	FALSE	FALSE	FALSE	FALSE
PQ2-30S_bin_14	1l, 1l, 1l, 4b, 4b, 4e, 4f, 4g	FALSE	FALSE	FALSE	FALSE	FALSE	FALSE	FALSE	FALSE	FALSE	FALSE	TRUE	FALSE	FALSE	FALSE	FALSE
PQ2-30S_bin_12	1l, 4b, 4g	TRUE	FALSE	FALSE	FALSE	TRUE	FALSE	FALSE	FALSE	FALSE	FALSE	FALSE	FALSE	FALSE	FALSE	FALSE
PQ2-30S_bin_9	4b, 4g	TRUE	FALSE	FALSE	FALSE	FALSE	FALSE	FALSE	FALSE	FALSE	FALSE	TRUE	FALSE	FALSE	FALSE	FALSE
PQ2-30S_bin_1	1h, 4g	FALSE	FALSE	FALSE	FALSE	FALSE	FALSE	FALSE	FALSE	TRUE	FALSE	TRUE	TRUE	FALSE	FALSE	FALSE
PQ2-30S_bin_5	1h, 4b, 4g	TRUE	FALSE	FALSE	FALSE	FALSE	FALSE	FALSE	FALSE	FALSE	FALSE	TRUE	FALSE	FALSE	FALSE	FALSE
PQ2-30S_bin_4	4e, 4f	TRUE	FALSE	FALSE	FALSE	FALSE	FALSE	FALSE	FALSE	TRUE	FALSE	FALSE	FALSE	FALSE	FALSE	TRUE
PQ2-30S_bin_16	4b, 4g	FALSE	FALSE	FALSE	FALSE	FALSE	FALSE	FALSE	FALSE	FALSE	FALSE	FALSE	FALSE	FALSE	FALSE	FALSE
PQ2-30S_bin_3	1h, 4b, 4f, 4g, 4g	TRUE	FALSE	FALSE	FALSE	FALSE	FALSE	FALSE	FALSE	TRUE	FALSE	TRUE	TRUE	FALSE	FALSE	FALSE
PQ2-30S_bin_6	1h, 1h, 1h, 4g	TRUE	FALSE	FALSE	FALSE	FALSE	FALSE	FALSE	FALSE	TRUE	FALSE	TRUE	TRUE	FALSE	FALSE	FALSE
PQ2-30S_bin_11	1h, 4e, 4f	TRUE	FALSE	FALSE	FALSE	FALSE	FALSE	FALSE	FALSE	FALSE	FALSE	FALSE	TRUE	FALSE	FALSE	FALSE
PQ2-30S_bin_10	1h, 4g, 4g	TRUE	FALSE	FALSE	FALSE	FALSE	FALSE	FALSE	FALSE	FALSE	FALSE	FALSE	FALSE	FALSE	FALSE	FALSE
PQ3-0S_bin_5	1b, 1b, 2d, 4e, 4g	FALSE	TRUE	TRUE	FALSE	TRUE	FALSE	TRUE	FALSE	FALSE	FALSE	FALSE	TRUE	TRUE	FALSE	FALSE
PQ3-0S_bin_17	4g, 4g	TRUE	FALSE	FALSE	FALSE	FALSE	FALSE	FALSE	FALSE	FALSE	FALSE	FALSE	FALSE	FALSE	FALSE	FALSE
PQ3-0S_bin_25	1e, 3b, 4e	FALSE	TRUE	TRUE	FALSE	FALSE	FALSE	FALSE	FALSE	FALSE	FALSE	TRUE	TRUE	FALSE	FALSE	FALSE
PQ3-0S_bin_10	4f, 4f, 4g	FALSE	FALSE	FALSE	FALSE	FALSE	FALSE	FALSE	FALSE	FALSE	FALSE	FALSE	TRUE	FALSE	FALSE	FALSE
PQ3-0S_bin_8	4g, 4g	FALSE	FALSE	FALSE	FALSE	FALSE	FALSE	FALSE	FALSE	FALSE	FALSE	FALSE	TRUE	FALSE	FALSE	FALSE
PQ3-0S_bin_15	4g	FALSE	TRUE	TRUE	FALSE	FALSE	FALSE	FALSE	FALSE	TRUE	FALSE	FALSE	TRUE	FALSE	FALSE	TRUE
PQ3-0S_bin_16	4b, 4b, 4f, 4g, 4g	FALSE	FALSE	FALSE	FALSE	FALSE	FALSE	FALSE	FALSE	TRUE	FALSE	FALSE	TRUE	FALSE	FALSE	FALSE
PQ3-0S_bin_24	4e, 4e, 4f, 4g	FALSE	FALSE	FALSE	FALSE	TRUE	FALSE	FALSE	FALSE	FALSE	FALSE	TRUE	TRUE	FALSE	FALSE	FALSE
PQ3-0S_bin_13	1e, 1e, 3b, 4g	FALSE	TRUE	TRUE	TRUE	TRUE	FALSE	FALSE	FALSE	TRUE	FALSE	TRUE	TRUE	FALSE	FALSE	FALSE
PQ3-0S_bin_2	4b, 4b, 4f, 4g, 4g	FALSE	FALSE	FALSE	FALSE	FALSE	FALSE	FALSE	FALSE	FALSE	FALSE	FALSE	TRUE	FALSE	FALSE	FALSE
PQ3-0S_bin_1	1b, 2d, 4e, 4g, 4g	FALSE	TRUE	FALSE	FALSE	TRUE	FALSE	TRUE	FALSE	FALSE	FALSE	FALSE	TRUE	TRUE	FALSE	FALSE
PQ3-0S_bin_12	1e, 1e, 2b, 3d, 4b, 4f, 4g	FALSE	TRUE	TRUE	TRUE	FALSE	FALSE	FALSE	FALSE	FALSE	FALSE	FALSE	TRUE	FALSE	FALSE	FALSE
PQ3-0S_bin_9	4b, 4f, 4f, 4g, 4g	FALSE	FALSE	FALSE	FALSE	FALSE	FALSE	FALSE	FALSE	FALSE	FALSE	FALSE	TRUE	FALSE	FALSE	FALSE
PQ3-0S_bin_7	4b, 4g, 4g	TRUE	FALSE	FALSE	FALSE	FALSE	FALSE	FALSE	FALSE	FALSE	FALSE	TRUE	TRUE	FALSE	FALSE	FALSE
PQ3-0S_bin_19	1f, 4b, 4g	TRUE	FALSE	FALSE	FALSE	FALSE	FALSE	FALSE	FALSE	FALSE	FALSE	TRUE	TRUE	FALSE	TRUE	FALSE
PQ3-0S_bin_14	1e, 3b, 4f, 4f, 4g	FALSE	FALSE	TRUE	TRUE	TRUE	FALSE	FALSE	FALSE	FALSE	FALSE	FALSE	TRUE	FALSE	FALSE	FALSE
PQ3-0S_bin_11	4e, 4f, 4f, 4g	FALSE	FALSE	FALSE	FALSE	FALSE	FALSE	FALSE	FALSE	FALSE	FALSE	FALSE	TRUE	FALSE	FALSE	FALSE
PQ3-0S_bin_27	3d, 4a, 4f, 4g, 4g, 4g, 4g	TRUE	FALSE	FALSE	FALSE	FALSE	FALSE	FALSE	FALSE	FALSE	FALSE	FALSE	FALSE	FALSE	FALSE	FALSE
PQ3-0S_bin_21	1b, 4b, 4e, 4g	TRUE	FALSE	FALSE	TRUE	FALSE	FALSE	FALSE	FALSE	FALSE	FALSE	FALSE	TRUE	FALSE	FALSE	FALSE
PQ3-0S_bin_4	3d, 4a, 4g, 4g	TRUE	FALSE	FALSE	FALSE	FALSE	FALSE	FALSE	FALSE	FALSE	FALSE	FALSE	FALSE	FALSE	FALSE	FALSE
PQ3-0S_bin_22	4f, 4h	FALSE	FALSE	FALSE	FALSE	FALSE	FALSE	FALSE	FALSE	FALSE	FALSE	FALSE	FALSE	FALSE	FALSE	FALSE
PQ3-0S_bin_18	3d, 4b, 4e, 4e, 4f, 4g	FALSE	FALSE	FALSE	FALSE	FALSE	FALSE	FALSE	FALSE	FALSE	FALSE	FALSE	TRUE	FALSE	FALSE	FALSE
PQ3-0S_bin_6	1f, 4b, 4f, 4f, 4g	FALSE	FALSE	FALSE	FALSE	FALSE	FALSE	FALSE	FALSE	FALSE	FALSE	FALSE	TRUE	FALSE	FALSE	FALSE
PQ3-0S_bin_3	4f, 4g, 4g, 4g	FALSE	FALSE	FALSE	FALSE	FALSE	FALSE	FALSE	FALSE	FALSE	FALSE	FALSE	FALSE	FALSE	FALSE	FALSE
PQ3-10S_bin_2	4e, 4f, 4f, 4g	FALSE	TRUE	FALSE	FALSE	TRUE	FALSE	FALSE	FALSE	TRUE	FALSE	FALSE	TRUE	FALSE	FALSE	TRUE
PQ3-10S_bin_5	1h, 4f, 4f, 4g	TRUE	FALSE	FALSE	FALSE	TRUE	FALSE	FALSE	FALSE	TRUE	FALSE	TRUE	TRUE	FALSE	FALSE	FALSE
PQ3-10S_bin_8	1h, 3b, 4g	TRUE	FALSE	FALSE	FALSE	TRUE	FALSE	FALSE	FALSE	TRUE	FALSE	FALSE	TRUE	FALSE	FALSE	FALSE
PQ3-10S_bin_7	4b, 4e, 4f, 4f, 4f, 4f, 4g	TRUE	TRUE	FALSE	FALSE	FALSE	FALSE	FALSE	FALSE	FALSE	FALSE	TRUE	TRUE	FALSE	FALSE	FALSE
PQ3-10S_bin_6	1h, 4f, 4g	FALSE	FALSE	FALSE	FALSE	FALSE	FALSE	FALSE	FALSE	FALSE	FALSE	TRUE	TRUE	FALSE	FALSE	FALSE
PQ3-10S_bin_13	4f, 4g	TRUE	TRUE	FALSE	FALSE	FALSE	FALSE	FALSE	FALSE	FALSE	FALSE	TRUE	TRUE	FALSE	FALSE	FALSE
PQ3-10S_bin_11	4f, 4g	TRUE	FALSE	FALSE	FALSE	FALSE	FALSE	FALSE	FALSE	FALSE	FALSE	FALSE	FALSE	FALSE	FALSE	FALSE
PQ3-10S_bin_9	1f, 3b, 4b, 4f, 4g, 4g	FALSE	TRUE	FALSE	FALSE	FALSE	FALSE	FALSE	FALSE	FALSE	FALSE	FALSE	TRUE	FALSE	FALSE	FALSE
PQ3-10S_bin_1	NULL	TRUE	FALSE	FALSE	FALSE	FALSE	FALSE	FALSE	FALSE	FALSE	FALSE	FALSE	TRUE	FALSE	FALSE	FALSE
PQ3-10S_bin_15	1d, 4b, 4e, 4f, 4f, 4f, 4g	FALSE	FALSE	FALSE	FALSE	TRUE	FALSE	FALSE	FALSE	FALSE	FALSE	TRUE	TRUE	FALSE	FALSE	FALSE
PQ3-10S_bin_3	4f	FALSE	FALSE	FALSE	FALSE	FALSE	TRUE	FALSE	FALSE	FALSE	FALSE	FALSE	FALSE	FALSE	FALSE	FALSE
PQ3-10S_bin_12	4f	FALSE	TRUE	FALSE	FALSE	TRUE	FALSE	FALSE	FALSE	FALSE	FALSE	TRUE	TRUE	FALSE	FALSE	TRUE
PQ3-10S_bin_4	4b, 4f, 4f, 4g	FALSE	TRUE	FALSE	FALSE	FALSE	FALSE	FALSE	FALSE	TRUE	FALSE	TRUE	TRUE	FALSE	FALSE	FALSE
PQ3-10S_bin_14	4g	FALSE	FALSE	FALSE	FALSE	FALSE	FALSE	FALSE	FALSE	FALSE	FALSE	FALSE	TRUE	FALSE	FALSE	FALSE
PQ3-20S_bin_2	4e	TRUE	FALSE	FALSE	FALSE	FALSE	TRUE	TRUE	FALSE	FALSE	FALSE	TRUE	TRUE	FALSE	FALSE	FALSE
PQ3-20S_bin_1	1e, 4g	TRUE	FALSE	FALSE	FALSE	FALSE	TRUE	TRUE	FALSE	FALSE	FALSE	FALSE	TRUE	FALSE	FALSE	FALSE
PQ3-20S_bin_3	4c, 4e	FALSE	FALSE	FALSE	FALSE	FALSE	FALSE	FALSE	FALSE	FALSE	FALSE	FALSE	FALSE	FALSE	FALSE	FALSE
PQ3-30S_bin_6	NULL	TRUE	FALSE	FALSE	FALSE	FALSE	FALSE	TRUE	FALSE	TRUE	FALSE	TRUE	TRUE	FALSE	FALSE	FALSE
PQ3-30S_bin_5	NULL	TRUE	FALSE	FALSE	FALSE	FALSE	FALSE	FALSE	FALSE	TRUE	FALSE	TRUE	TRUE	FALSE	FALSE	FALSE
PQ3-30S_bin_4	1l	TRUE	FALSE	FALSE	FALSE	TRUE	FALSE	FALSE	FALSE	TRUE	FALSE	TRUE	TRUE	FALSE	FALSE	FALSE
PQ3-30S_bin_12	1d, 4c, 4e	FALSE	FALSE	FALSE	FALSE	FALSE	FALSE	FALSE	FALSE	FALSE	FALSE	TRUE	TRUE	FALSE	FALSE	FALSE
PQ3-30S_bin_2	4b	TRUE	FALSE	FALSE	FALSE	FALSE	FALSE	FALSE	TRUE	TRUE	FALSE	TRUE	FALSE	FALSE	FALSE	FALSE
PQ3-30S_bin_8	4g	FALSE	FALSE	FALSE	FALSE	TRUE	FALSE	FALSE	FALSE	FALSE	FALSE	TRUE	TRUE	FALSE	FALSE	TRUE
PQ3-30S_bin_9	4b	TRUE	FALSE	FALSE	FALSE	FALSE	FALSE	FALSE	FALSE	TRUE	FALSE	TRUE	TRUE	FALSE	FALSE	FALSE
PQ3-30S_bin_10	4b, 4b, 4g, 4g	FALSE	FALSE	FALSE	FALSE	FALSE	FALSE	FALSE	FALSE	FALSE	FALSE	FALSE	FALSE	FALSE	FALSE	FALSE
PQ3-30S_bin_3	1h, 3d, 4e, 4f, 4f	TRUE	FALSE	FALSE	FALSE	FALSE	FALSE	FALSE	FALSE	FALSE	FALSE	FALSE	TRUE	FALSE	FALSE	FALSE
PQ3-30S_bin_7	4e	TRUE	FALSE	FALSE	FALSE	FALSE	TRUE	FALSE	FALSE	FALSE	FALSE	FALSE	FALSE	FALSE	FALSE	FALSE
PQ3-30S_bin_11	NULL	FALSE	TRUE	FALSE	FALSE	FALSE	FALSE	FALSE	FALSE	FALSE	FALSE	FALSE	TRUE	FALSE	FALSE	FALSE
PQ3-30S_bin_13	NULL	TRUE	FALSE	FALSE	FALSE	FALSE	TRUE	FALSE	FALSE	FALSE	FALSE	FALSE	FALSE	FALSE	FALSE	FALSE
PQ2-0S_bin_16	NULL	FALSE	FALSE	FALSE	FALSE	FALSE	FALSE	FALSE	FALSE	FALSE	FALSE	FALSE	FALSE	FALSE	FALSE	FALSE
PQ2-0S_bin_9	NULL	FALSE	FALSE	FALSE	FALSE	FALSE	FALSE	FALSE	FALSE	FALSE	FALSE	FALSE	FALSE	FALSE	FALSE	FALSE
PQ2-20S_bin_8	NULL	FALSE	FALSE	FALSE	FALSE	FALSE	FALSE	FALSE	FALSE	FALSE	FALSE	FALSE	FALSE	FALSE	FALSE	FALSE
PQ3-0S_bin_20	NULL	FALSE	FALSE	FALSE	FALSE	FALSE	FALSE	FALSE	FALSE	FALSE	FALSE	FALSE	FALSE	FALSE	FALSE	FALSE
PQ3-0S_bin_26	NULL	FALSE	FALSE	FALSE	FALSE	FALSE	FALSE	FALSE	FALSE	FALSE	FALSE	FALSE	FALSE	FALSE	FALSE	FALSE
PQ3-0S_bin_28	4f, 4g, 4g	TRUE	FALSE	FALSE	FALSE	FALSE	FALSE	FALSE	FALSE	FALSE	FALSE	FALSE	FALSE	FALSE	FALSE	FALSE

[a] Presence of metabolic pathways confirmed via the criteria described in Rogers et al. 2023 (ISME) and Supplemental Methods

[b] [NiFe]-Hydrogenase subgroups present in MAG; color-coded for Group 1 subgroups (red: 1a, orange: 1b, blue: 1d, yellow: 1e, gray: 1f, green: 1h and 1l; not detected: 1c, 1g, 1i, 1j, 1k)

[c] Carbon Monoxide Dehydrogenase (CoxL)

Confirmed "TRUE" pathways are highlighted in green

KEGG modules (M00xxx) and abbreviations: CBB (Calvin-Benson-Bassham; M00165), WL (Wood-Ljungdahl; M00377), rTCA (Reductive TCA; M00173), 3-HPB (3-Hydroxypropionate Bicycle; M00376), HP-HB (Hydroxypropionate-Hydroxybutylate Cycle; M00375), DC-HB (Dicarboxylate-Hydroxybutyrate Cycle; M00374), DSR (Dissimilatory Sulfate Reduction; M00596), Thio Ox (Thiosulfate Oxidation; M00595), PS I/II (Photosystem I/II; M00161/M00163), Denit (Denitrification; M00529), DNR (Dissimilatory Nitrate Reduction; M00530), Met Gen + substrate (Methanogenesis; M00567, M00357, M00356, M00563), Met Ox (Methane Oxidation; M00174)

References

1. Vuillemin A, Wankel SD, Coskun ÖK, Magritsch T, Vargas S, Estes ER, et al. Archaea dominate oxic subseafloor communities over multimillion-year time scales. 2019; **5**: eaaw4108.
2. Krueger F, James F, Ewels P, Afyounian E, Weinstein M, Schuster-Boeckler B, et al. TrimGalore. 2023. Zenodo.
3. Hannon GJ. FASTX-Toolkit. 2010.
4. Aramaki T, Blanc-Mathieu R, Endo H, Ohkubo K, Kanehisa M, Goto S, et al. KofamKOALA: KEGG Ortholog assignment based on profile HMM and adaptive score threshold. *Bioinformatics* 2020; **36**: 2251–2252.
5. Leung PM, Greening C. Compiled Greening lab metabolic marker gene databases. 2021. Monash University.
6. Li W, Godzik A. Cd-hit: a fast program for clustering and comparing large sets of protein or nucleotide sequences. *Bioinformatics* 2006; **22**: 1658–1659.
7. Katoh K, Standley DM. MAFFT multiple sequence alignment software version 7: improvements in performance and usability. *Mol Biol Evol* 2013; **30**: 772–780.
8. Darriba D, Posada D, Kozlov AM, Stamatakis A, Morel B, Flouri T. ModelTest-NG: a new and scalable tool for the selection of DNA and protein evolutionary models. *Mol Biol Evol* 2020; **37**: 291–294.
9. Kozlov AM, Darriba D, Flouri T, Morel B, Stamatakis A. RAxML-NG: a fast, scalable and user-friendly tool for maximum likelihood phylogenetic inference. *Bioinformatics* 2019; **35**: 4453–4455.
10. Tassi F, Aguilera F, Darrah T, Vaselli O, Capaccioni B, Poreda RJ, et al. Fluid geochemistry of hydrothermal systems in the Arica-Parinacota, Tarapacá and Antofagasta regions (northern Chile). *J Volcanol Geotherm Res* 2010; **192**: 1–15.

11. Lindsay MR, Colman DR, Amenabar MJ, Fristad KE, Fecteau KM, Debes R V., et al. Probing the geological source and biological fate of hydrogen in Yellowstone hot springs. *Environ Microbiol* 2019; **21**: 3816–3830.
12. Islam ZF, Cordero PRF, Feng J, Chen Y-J, Bay SK, Jirapanjawat T, et al. Two Chloroflexi classes independently evolved the ability to persist on atmospheric hydrogen and carbon monoxide. *ISME J* 2019; **13**: 1801–1813.
13. Weber CF, King GM. Distribution and diversity of carbon monoxide-oxidizing bacteria and bulk bacterial communities across a succession gradient on a Hawaiian volcanic deposit. *Environ Microbiol* 2010; **12**: 1855–1867.
14. King CE, King GM. Description of *Thermogemmatispora carboxidivorans* sp. nov., a carbon-monoxide-oxidizing member of the class *Ktedonobacteria* isolated from a geothermally heated biofilm, and analysis of carbon monoxide oxidation by members of the class *Ktedonobacteria*. *Int J Syst Evol Microbiol* 2014; **64**: 1244–1251.
15. Ghezzi D, Sauro F, Columbu A, Carbone C, Hong P-Y, Vergara F, et al. Transition from unclassified *Ktedonobacterales* to *Actinobacteria* during amorphous silica precipitation in a quartzite cave environment. *Sci Rep* 2021; **11**: 3921.
16. Lynch RC, King AJ, Farías ME, Sowell P, Vitry C, Schmidt SK. The potential for microbial life in the highest-elevation (>6000 m.a.s.l.) mineral soils of the Atacama region. *J Geophys Res Biogeosciences* 2012; **117**: G02028.
17. Jordaan K, Lappan R, Dong X, Aitkenhead IJ, Bay SK, Chiri E, et al. Hydrogen-oxidizing bacteria are abundant in desert soils and strongly stimulated by hydration. *mSystems* 2020; **5**: e01131-20.
18. Ray AE, Zhang E, Terauds A, Ji M, Kong W, Ferrari BC. Soil microbiomes with the genetic capacity for atmospheric chemosynthesis are widespread across the poles and are associated with moisture, carbon, and nitrogen limitation. *Front Microbiol* 2020; **11**: 1936.
